# Musculoskeletal and body composition response to high-dose testosterone with finasteride after chronic incomplete spinal cord injury—a randomized, double-blind, and placebo-controlled pilot study

**DOI:** 10.3389/fneur.2024.1479264

**Published:** 2024-12-11

**Authors:** Dana M. Otzel, Larissa Nichols, Christine F. Conover, Stephen A. Marangi, Jayachandra R. Kura, Dominic K. Iannaccone, David J. Clark, Chris M. Gregory, Christopher F. Sonntag, Anita Wokhlu, Hans K. Ghayee, Michael J. McPhaul, Charles E. Levy, Charles A. Plumlee, Robert B. Sammel, Kevin T. White, Joshua F. Yarrow

**Affiliations:** ^1^Brain Rehabilitation Research Center, Malcom Randall Department of Veterans Affairs Medical Center, North Florida/South Georgia Veterans Health System, Gainesville, FL, United States; ^2^Department of Physiology & Aging, University of Florida College of Medicine, Gainesville, FL, United States; ^3^Department of Neurology, University of Florida College of Medicine, Gainesville, FL, United States; ^4^Department of Health Sciences and Research, Medical University of South Carolina, Charleston, SC, United States; ^5^Diagnostic Imaging Service – Radiology, Malcom Randall Department of Veterans Affairs Medical Center, North Florida/South Georgia Veterans Health System, Gainesville, FL, United States; ^6^Medical Specialties Service – Cardiology, Malcom Randall Department of Veterans Affairs Medical Center, North Florida/South Georgia Veterans Health System, Gainesville, FL, United States; ^7^Division of Cardiovascular Medicine, Department of Medicine, University of Florida College of Medicine, Gainesville, FL, United States; ^8^Division of Endocrinology, Diabetes, and Metabolism, Department of Medicine, University of Florida College of Medicine, Gainesville, FL, United States; ^9^Quest Diagnostics Nichols Institute, San Juan Capistrano, CA, United States; ^10^Physical Medicine and Rehabilitation Service, Malcom Randall Department of Veterans Affairs Medical Center, North Florida/South Georgia Veterans Health System, Gainesville, FL, United States; ^11^Spinal Cord Injury Service, Malcom Randall Department of Veterans Affairs Medical Center, North Florida/South Georgia Veterans Health System, Gainesville, FL, United States; ^12^Geriatrics and Extended Care, South Texas Veterans Health Care System, Kerrville, TX, United States; ^13^Michael Bilirakis VA Spinal Cord Injury/Disorders Center, James A. Haley Department of Veterans Affairs Medical Center, Tampa, FL, United States; ^14^Eastern Colorado Geriatrics Research, Education, and Clinical Center, Rocky Mountain Regional Department of Veterans Affairs Medical Center, VA Eastern Colorado Health Care System, Aurora, CO, United States; ^15^Division of Geriatric Medicine, Department of Medicine, University of Colorado Anschutz Medical Campus, Aurora, CO, United States

**Keywords:** androgen, estrogen, testosterone replacement, hypogonadism, spinal cord injury, muscle, bone, fat

## Abstract

**Background:**

High-dose testosterone replacement therapy (TRT), paired with finasteride (type II 5α-reductase inhibitor), improves body composition, muscle strength, and bone mineral density (BMD) in older men, without inducing prostate enlargement—a side effect associated with TRT. Men with spinal cord injury (SCI) exhibit neuromuscular impairment, muscle atrophy, bone loss, and increased central adiposity, along with low testosterone. However, sparse evidence supports TRT efficacy after SCI.

**Methods:**

This parallel-group, double-blind, placebo-controlled, and randomized clinical trial (RCT) is a pilot study that enrolled men (*N* = 12) with low to low–normal testosterone and gait impairments after chronic motor-incomplete SCI. Participants received high-dose intramuscular TRT (testosterone-enanthate, 125 mg/week) with finasteride (5 mg/day) vs. vehicle+placebo for 12 months. Change relative to baseline was determined for body composition, musculoskeletal outcomes, and prostate size, with effect sizes calculated between groups using Hedges’ *g*. Adverse events and feasibility were assessed.

**Results:**

TRT + finasteride consistently increased testosterone (*g* = 1.16–3.08) and estradiol (*g* = 0.43–3.48), while concomitantly reducing dihydrotestosterone (*g* = 0.31–2.27). Very large effect sizes at both 6 and 12 months suggest TRT + finasteride increased whole-body fat-free (lean) mass (+3–4% vs. baseline, *g* = 2.12–2.14) and knee extensor (KE) whole-muscle cross-sectional area (+8–11% vs. baseline, *g* = 2.06–2.53) more than vehicle+placebo. Moderate-to-large effect sizes suggest TRT + finasteride increased KE maximal voluntary isometric torque (+15–40% vs. baseline, *g* = 0.47–1.01) and femoral neck and distal femur BMD from 6 months onward (*g* = 0.51–1.13), compared with vehicle+placebo, and reduced fat mass 9–14% within the whole-body, trunk, and android (visceral) regions at 12 months (*g* = 0.77–1.27). TRT + finasteride also produced small effect sizes favoring lesser prostate growth than vehicle+placebo (*g* = 0.31–0.43). The participant retention, drug compliance, and incidence and severity of adverse events were similar among the groups.

**Conclusion:**

These data provide proof-of-concept and rationale for larger RCTs aimed at discerning the impact of TRT + finasteride on body composition, musculoskeletal health, and physical function in men with SCI, along with effect sizes and variance of responses to assist in planning subsequent trials.

**Clinical trial registration:**

ClinicalTrials.gov, identifier NCT02248701.

## 1 Introduction

Spinal cord injury (SCI) impairs neuromuscular function below the spinal lesion, typically resulting in muscle atrophy (1), bone loss, and high fracture risk (2). These neuromusculoskeletal deficits limit voluntary locomotor capacity in the impaired limbs and may lower basal metabolic rate (3), predisposing to visceral fat accumulation and a sequelae of metabolic consequences (4) resulting from low energy expenditure. A host of secondary endocrine changes also accompany SCI, including low testosterone (5), which has the potential to hasten the musculoskeletal decline resulting from SCI (6) and to increase fat gain and worsen cardiometabolic health (7). Indeed, the majority of the men with SCI exhibit low testosterone throughout the initial 12 months postinjury (8, 9) and roughly 20–50% of men with SCI display persistently low testosterone (10–12). However, the influence of low testosterone on muscle atrophy, bone loss, and fat accumulation in men with SCI remains understudied, as does the ability of testosterone replacement therapy (TRT) to mitigate these adverse effects of SCI (13).

In men without neurologic injury, low testosterone is associated with low muscle mass and low bone mineral density (BMD), and with increased adiposity (14) and deleterious metabolic outcomes (15). However, inconsistency exists in the literature regarding TRT efficacy across randomized clinical trials (RCTs), with some studies reporting minimal musculoskeletal and body composition benefits and others reporting more substantial improvement (16). For example, several RCTs that administered transdermal TRT to older men without neurologic injury reported small increases in fat-free (lean) mass, ranging from 1–2 kg over 12–36 months of treatment, but no detectable increase was evident in lower extremity muscle strength when compared with placebo (17–22). Other RCTs that provided intramuscular testosterone-enanthate observed a 3–4 kg increase in lean mass after only 5–12 months and an accompanying ~10–25% increase in the lower extremity muscle strength (23–25). Similarly, meta-analyses report that intramuscular TRT produced, on average an approximately 3.5 times greater increase in lean mass than transdermal TRT formulations, along with more significant increases in muscle strength (26) and BMD (27), when compared with their respective placebos.

TRT is also associated with several side effects, the most common of which are increased risks of polycythemia, reduced high-density lipoprotein (HDL) cholesterol, and combined prostate/lower urinary tract-related events (28). Meta-analyses have confirmed that intramuscular TRT increased prostate-specific antigen (PSA) more so than transdermal TRT formulations (29, 30) and that TRT is associated with an increase in prostate volume (31), independent of other prostate events. However, these prostate events are driven by the intraprostatic conversion of testosterone to dihydrotestosterone via the 5α-reductase isozymes and not directly by TRT (16). In this regard, our laboratory (25) and others (32, 33) have reported that finasteride, a type II 5α-reductase inhibitor, reduced circulating dihydrotestosterone by 50–75% and prevented the increase in PSA and prostate growth associated with high-dose intramuscular testosterone-enanthate without inhibiting the beneficial body composition and musculoskeletal changes that result from TRT.

No consensus exists regarding the ability of TRT to improve musculoskeletal health or body composition in the SCI population because, to date, no double-blind, placebo-controlled RCT has assessed TRT efficacy in men with SCI. Indeed, the results of only two small prospective trials exist on this topic in the literature, both open-label, non-placebo-controlled studies (34, 35). In the first, Bauman et al. provided moderate-dose transdermal TRT to men who exhibited low testosterone after chronic motor-complete SCI and observed increased lean mass within the whole body, trunk, and paralyzed lower extremities, along with a concomitant increase in resting metabolic rate (34). In comparison, several studies from the second trial reported that low-dose transdermal TRT did not improve body composition nor basal metabolic rate (35), muscle cross-sectional area (CSA), muscle force production (36–38), nor bone microstructure (39) in men with chronic motor-complete SCI. While neither trial utilized intramuscular TRT, studies from our laboratory demonstrated that the high-dose intramuscular testosterone-enanthate attenuated muscle atrophy and trabecular bone loss that developed in the paralyzed limbs in a rodent severe SCI model (40–43). Moreover, in this rodent SCI model, co-administration of finasteride completely prevented prostate enlargement resulting from high-dose intramuscular TRT without inhibiting these musculoskeletal benefits (41). As such, well-controlled RCTs are needed to determine whether intramuscular TRT paired with finasteride (TRT + finasteride) can safely improve body composition and musculoskeletal health in men with low testosterone after SCI.

The primary purpose of this pilot RCT study was to determine effect sizes and variance of responses for body composition and musculoskeletal outcomes in response to TRT + finasteride vs. vehicle+placebo in men with low to low–normal testosterone and impaired gait after chronic motor-incomplete SCI. Efficacy-based outcomes were whole-body, upper body, and lower extremity fat-free (lean) mass; whole-body, trunk, and android (visceral) fat mass; knee extensor (KE) muscle cross-sectional area (CSA) and maximal voluntary isometric contraction (MVIC); total hip, femoral neck, lumbar spine, and distal femur areal (a) BMD; and circulating bone resorption and formation markers. The secondary purposes were to obtain effect sizes and variance of response for change in prostate volume and initial estimates comparing the prevalence of other adverse events (AE).

## 2 Materials and methods

### 2.1 Study design

This parallel-group, double-blind, and placebo-controlled pilot RCT was reviewed and approved by the U.S. Department of Veterans Affairs Central Institutional Review Board (VA CIRB, Washington, D.C., United States), authorized under the U.S. Food and Drug Administration (FDA) Investigational New Drug No.123911, registered at clinicaltrials.gov (NCT02248701), and conducted at North Florida/South Georgia Veterans Health System (Gainesville, FL, United States) and Michael Bilirakis VA SCI Center at James A. Haley Veterans’ Hospital (Tampa, FL, United States).

**Figure 1:**
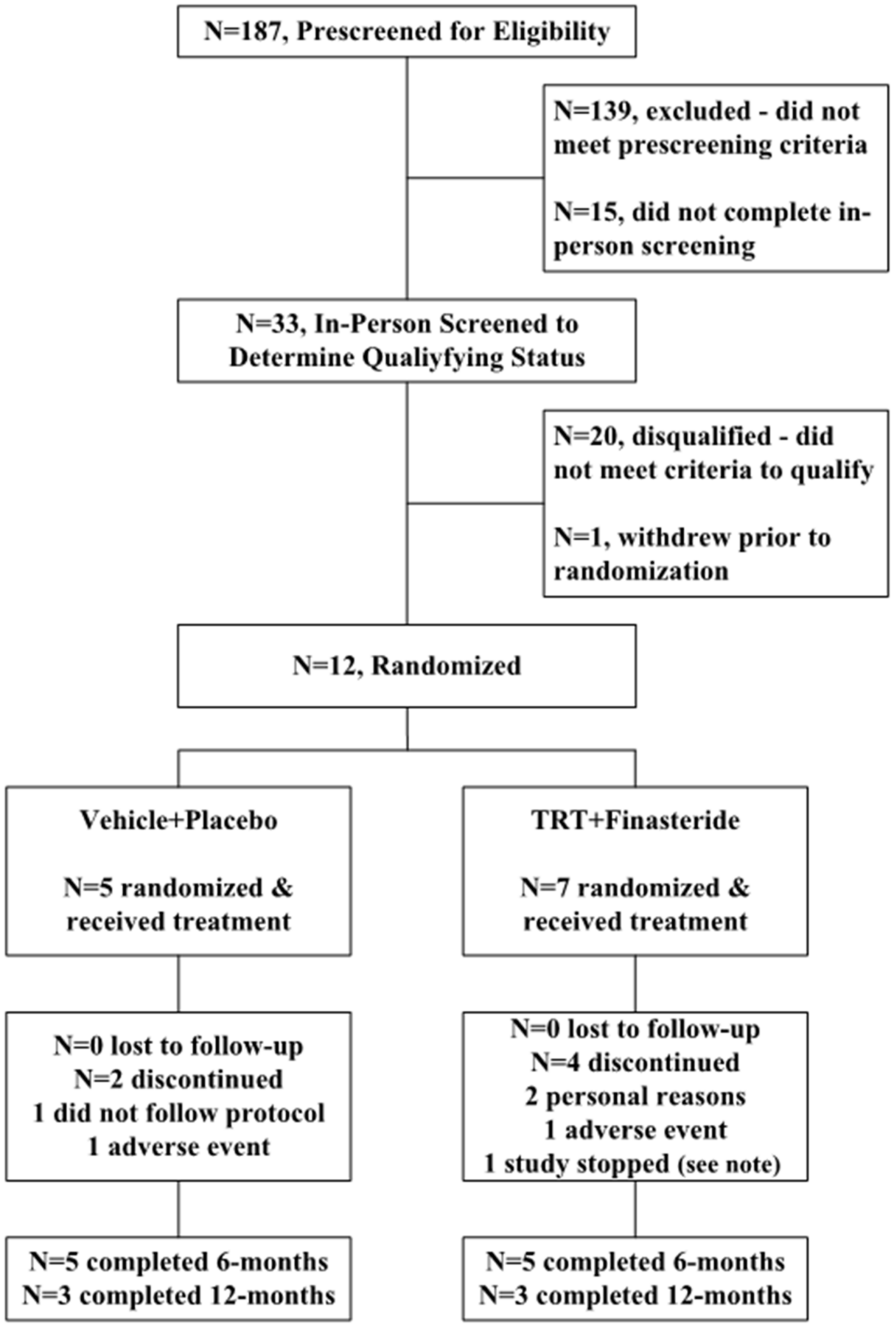
Study flow for participant enrollment, randomization, and completion. Note: Study enrollment was stopped for *N* = 1 participant in the testosterone replacement therapy plus finasteride (TRT + finasteride) group due to the Veterans Health Administration nationwide halt on all in-person research visits due to the SARS-CoV-2 (COVID-19) pandemic.

The participants were recruited from VA Medical Centers, outpatient clinics, and non-VA settings during 2017–2021. Interested persons were prescreened via telephone to determine if they met enrollment criteria (Figure 1). Those who enrolled provided written informed consent to participate in the study and underwent in-person screening to determine eligibility, including a structured medical history, questionnaires assessing physician-prescribed and over-the-counter medication use, fasting blood acquisition to determine total testosterone, bioavailable testosterone, and other circulating markers of health, a comprehensive physical examination with electrocardiogram to assess cardiac abnormalities, prostate digital rectal exam (DRE) to assess prostate abnormalities, transrectal ultrasound sizing (TRUS) to determine prostate volume, and 10-meter walking tests (10-m WT) to assess gait parameters. Other assessments were performed to classify baseline functional status and physical activity participation, including Modified Ashworth Scale for lower limb spasticity (44), Spinal Cord Independence Measure (SCIM) III (45), Berg Balance Scale (46), Walking Index for Spinal Cord Injury (WISCI) II (47), Craig Handicap Assessment and Reporting Technique Short Form (CHART SF) Questionnaire (48), Veteran Rand 12-Item Health Survey (VR-12) (49), and Stanford 7-Day Activity Recall (50).

### 2.2 Study participants

The participants in our study were medically stable men, ≥18 years of age, who had experienced a motor-incomplete SCI [American Spinal Injury Association Impairment Scale (AIS) C/D] between spinal levels C2–L3 with upper motor neuron signs that resulted from trauma, vascular, or orthopedic pathology >12 months prior. The participants qualified for the study if they met the aforementioned criteria and exhibited low to low–normal total testosterone (≤325 ng/dL) and/or bioavailable testosterone (≤70 ng/dL) and ambulatory dysfunction, defined as self-selected gait speed between 0.10–1.30 m/s on 10-m WT and/or visibly impaired gait parameters without exclusionary criteria.

For this study, we excluded participants whose life expectancy was below 12 months and those with a history of congenital SCI (e.g., Chiari malformation, myelomeningocele, intraspinal neoplasm, and Frederich’s ataxia), or degenerative spinal disorder (e.g., spinocerebellar degeneration and syringomyelia) that would complicate study procedures or data interpretation; multiple sclerosis, amyotrophic lateral sclerosis, or other neurologic impairment/injury; any poorly compensated or uncontrolled cardiovascular disease; venous thromboembolism within the last 6 months, including deep venous thromboembolism and pulmonary embolism, history of recurrent venous thromboembolism or known hereditary thrombophilia; any major cardiovascular event within the last 12 months, defined as a history of acute myocardial infarction, any cardiac revascularization procedure including angioplasty, stenting, or coronary artery bypass grafting, hospitalization due to unstable angina, transient ischemic attack, or stroke; angina not controlled on a current medical regimen (Canadian class II, class III, or class IV); New York Heart Association (NYHA) class III or IV congestive heart failure; consistently measured systolic blood pressure ≥ 160 mm Hg or diastolic blood pressure ≥ 100 mm Hg; poorly controlled arrythmia; severe valvular disease; low-density lipoprotein (LDL) cholesterol >160 mg/dL with known history of any major cardiovascular event within the last 12 months; baseline echocardiogram findings (e.g., left bundle branch block) or marked echocardiogram abnormalities that would preclude serial screening for occult ischemic events; current prostate, breast or other organ cancer or history of cancer, except for completely resolved basal or squamous cell carcinoma or melanoma for a duration >24 months; benign prostate enlargement, defined as prostate volume > 40 mL evaluated via TRUS; serum PSA >3.0 ng/mL; hematocrit >49%; liver enzymes [alanine transaminase (ALT) or aspartate aminotransferase (AST)] above reference range upper limits; creatinine >1.4 mg/dL; calcium >10.5 mg/dL; diagnosed gynecomastia; diagnosed but untreated moderate or severe sleep apnea; high risk malnutrition (>15 on Spinal Nutrition Screening Tool) (51); severe claustrophobia that precluded magnetic resonance imaging (MRI); current anticoagulant therapy precluding intramuscular injections; use of any of the following agents that alter sex-steroid metabolism in the previous 3 months: testosterone, leuprolide, androgenic hormones, growth hormone, oral androgen precursors, 5α-reductase inhibitors, or aromatase inhibitors; use of antiresorptive or bone anabolic drug therapy in the previous 6 months; known allergy to sesame oil (vehicle); active participation in another research protocol that may influence study outcomes; or mental state that precluded understanding the study protocol.

### 2.3 Study procedures

Qualifying participants were stratified by walking speed (≤0.50 m/s vs. >0.50 m/s) and randomized 1:1 using RanPro (Applied Logic Associations, Houston, TX, United States) by the VA Research Pharmacy (Gainesville, FL, United States) to receive testosterone-enanthate (TRT, 125 mg/week, i.m.) plus finasteride (5 mg/day, p.o.) vs. vehicle [sterile sesame oil with chlorobutanol (preservative), 1 mL/week, i.m.] plus placebo (5 mg/day, p.o.) for 12 months. The drugs were purchased by the VA Research Pharmacy (Gainesville, FL, United States) from a commercial vendor. An accredited compounding pharmacy (Westlab Pharmacy, Gainesville, FL, United States) encapsulated finasteride and placebo to ensure identical capsule size, weight, appearance, and internal consistency and compounded the vehicle to match consistency, color, and other TRT characteristics, with sterility verified by Analytical Research Laboratories (Oklahoma City, OK, United States). Drugs and matching vehicle/placebo were distributed in identical packaging to ensure double-blind procedures were maintained. The participants were administered medications at each visit and were instructed on at-home drug administration. Drug compliance was determined via weekly telephone calls, by counting remaining finasteride or placebo capsules at each visit, and by direct assessment of circulating testosterone and dihydrotestosterone performed at 1–3-months intervals to verify drug delivery (described in the following). The VA Research Pharmacy adjusted TRT doses in 25 mg/week increments to maintain nadir testosterone between 400 ng/dL and 869 ng/dL in TRT + finasteride, and randomly adjusted vehicle doses to maintain double blinding.

### 2.4 Outcomes

The primary body composition, muscle, and bone outcomes were whole-body fat mass, KE whole-muscle CSA, and total hip areal bone mineral density (aBMD), respectively. Secondary outcomes were visceral fat mass, KE MVIC, and lumbar spine aBMD. Other exploratory outcomes included: whole-body, upper body, and lower body lean mass; trunk fat mass; femoral neck and distal femur aBMD; circulating CTX-1 (bone resorption marker), TRAcP 5b (osteoclast-derived bone resorption marker), and osteocalcin (osteoblast-derived bone formation marker); and prostate volume. Efficacy outcomes were assessed at baseline, with imaging assessments occurring at 6-months intervals and other assessments at 3-months intervals. Other blood analyses and safety assessments are described in the following.

#### 2.4.1 Imaging assessments

The participants arrived after an overnight fast and were placed supine for 30 min before scans. The participant’s legs were strapped together to limit movement due to involuntary muscle spasms, with the non-dominant limb assessed except when metal hardware precluded accurate muscle or bone imaging/analysis. A registered MRI technologist (VA Diagnostic Imaging Service, Gainesville, FL, United States) performed lower-extremity MRI scans using a standard body coil in a 1.5 Tesla magnet (SIGNA™ Artist, Chicago, IL, United States) per clinical guidelines. An average of 29 transaxial images were acquired (fast spin echo, TR = 622–647 ms, TE = 9 ms, TL = 4, flip angle = 30°, field of view = 42 cm, matrix size = 320 × 256, slice thickness = 4 mm, interslice distance =1 mm) from 6 in above the knee joint toward the hip. Acquisition time was <5 min. A region of interest encompassing the KE whole muscle was manually segmented by a single operator (DKI) using ImageJ (NIH, Bethesda, MD, United States). A licensed radiologic technologist (VA Diagnostic Imaging Service, Gainesville, FL, United States) assessed aBMD and body composition using a Hologic Discovery A Dual-Energy X-ray absorptiometry (DXA) system (Marlborough, MA, United States) that was calibrated daily with multistage hydroxyapatite and soft-tissue phantoms (CV < 1%). The whole-body, hip, and lumbar (L2–L4) spine scans were conducted per International Society for Clinical Densitometry guidelines (52). The distal femur was imaged using an established protocol (53) with the knee stabilized in full extension at 0° internal rotation against a 90° support beam with aBMD determined in a region of interest (ROI), starting 15% from the distal end of the femur and spanning 5% of the total femur length in the proximal direction. All scans were reviewed by a musculoskeletal radiologist (CFS).

#### 2.4.2 Functional assessments

Functional assessments were overseen by a licensed athletic trainer (DMO). Before testing, participants underwent a standardized warm-up and familiarization protocol. Ambulatory status was assessed during repeated 10-m WT, per standard protocol (54). A calibrated dynamometer (Biodex, Shirley, NY, United States) assessed KE MVIC at 60° knee flexion. Isometric contractions were used to ensure minimal impact of lower extremity spasticity on torque measurements. During the assessments, the participants were instructed to contract their muscles as forcefully as possible and hold the contraction for up to 5 s, with verbal encouragement provided throughout.

#### 2.4.3 Blood analysis

Fasting blood samples were acquired twice between 07:00 and 10:00 a.m. at baseline, and 1 week following the last drug or vehicle injection at all subsequent timepoints and evaluated for total, bioavailable, and free testosterone; dihydrotestosterone; estradiol; sex hormone binding globulin (SHBG); complete blood count (including hematocrit and hemoglobin); comprehensive metabolic panel; lipid panel; PSA; CTX-1; TRAcP 5b; osteocalcin; and other health markers (Supplementary Table S1). Total testosterone was measured by automated Cobas® electrochemiluminescence analysis (Roche Diagnostics, Indianapolis, IN, United States), a clinical standard in the VA Pathology and Laboratory Medicine Service (PLMS), and was verified via liquid chromatography–tandem mass spectrometry (LC–MS/MS, Quest Diagnostics Nichols Institute, San Juan Capistrano, CA, United States). Bioavailable testosterone was calculated per our methods (55). Free testosterone (calculated), dihydrotestosterone, and estradiol were determined with LC–MS/MS, SHBG via immunoassay (Quest Diagnostics), and CTX-1, TRAcP 5b, and osteocalcin by ELISA (Immunodiagnostic Systems, Ltd., Tyne and Wear, UK). Other measures were assessed within VA PLMS per clinical standards.

#### 2.4.4 Safety monitoring

Data on AEs were collected via systematic assessment at study visits and during weekly telephone assessments, with AE classified as non-serious or serious (SAE) using *a priori* criteria. Systemically assessed non-serious AE were considered laboratory values outside standard reference ranges regardless of timepoint or other non-life-threatening, non-serious events. SAE was defined by the VA CIRB (Washington., D.C., United States) and the FDA guidelines. The safety monitoring was overseen by the study physicians (RBS, CEL, CAP, and AW) and medical director (KTW) and included assessments of blood markers; comprehensive physical exams, including prostate DRE, prostate sizing via TRUS (VA Urology Service, Gainesville, FL, United States), and echocardiograms (VA Cardiology Service, Gainesville, FL, United States); and review of all non-serious AE and SAE.

*A priori* withdrawal criteria included development of prostate, breast, or other organ cancer; gynecomastia; severe peripheral edema classified as 2+ or higher; any adverse cardiovascular event, including acute myocardial infarction, acute coronary syndrome, new onset congestive heart failure, newly diagnosed flow-limiting coronary artery disease, cardiovascular-related hospitalization, stroke, or cardiac arrest, any new significant ischemic findings or symptoms, including development of new major abnormalities on serial echocardiogram or symptoms of angina; or any other SAE. *A priori* temporary stopping criteria included detection of a prostate nodule via DRE; PSA >4.0 ng/mL or > 1.4 ng/mL increase vs. baseline; hematocrit >52% or hemoglobin >17.5 g/dL; AST or ALT >1.5 times reference range upper limit; or calcium between 10.5 and 11.2 mg/dL with symptoms of hypercalcemia or > 11.2 mg/dL. Laboratory assessments were repeated if aberrant values were detected, and participants were withdrawn if values were verified, except for elevated hematocrit, hemoglobin, PSA, or prostate nodule. For prostate nodules, participants were referred to the VA Urology Service (Gainesville, FL, United States) to undergo a prostate biopsy to assess for prostate cancer and could resume if a biopsy was negative. For elevated PSA, the participants were referred for lower urinary tract infection (UTI) assessment—common due to intermittent catheterization after SCI (56)—and were offered treatment if detected. For elevated hematocrit or hemoglobin, participants were reassessed 4 weeks after drug discontinuation. The participants could resume upon renormalization of hematocrit, hemoglobin, or PSA, with physician approval, but were withdrawn if elevated values persisted for >4 weeks, upon prostate cancer detection, or if testing was refused.

### 2.5 Statistical methods

Values are means ± standard deviation (SD), with efficacy outcomes reported as percentage change from baseline at 3 or 6 months intervals and clinical laboratory and safety measures reported as absolute change from baseline. Hedge’s *g*, which adjusts for differing sample sizes and small sample size bias (57), was used to determine the effect sizes between the groups, for each efficacy-based outcome at each timepoint, which were categorized as small (*g* = 0.20), medium (*g* = 0.50), large (*g* = 0.80), or very large (*g* = 1.30) (58), and reported only when the values met these thresholds. Our initial plan was to complete *N* = 10/group. However, this study was stopped prematurely due to enrollment difficulties that resulted, in part, from the emergence of the severe acute respiratory syndrome coronavirus 2 (SARS-CoV-2) (COVID-19) pandemic and a resulting VA-mandated nationwide halt on in-person research. To account for low enrollment, results are considered preliminary data that require confirmation in larger RCTs.

## 3 Results

### 3.1 Baseline characteristics

Twelve men passed the screening criteria and were randomized into TRT + finasteride (*N* = 7) and vehicle+placebo (*N* = 5) groups. The participants within each group had similar SCI characteristics (Table 1) and roughly comparable baseline values for outcome measures (Table 2), although KE whole-muscle CSA and KE MVIC were directionally higher in TRT + finasteride vs. vehicle+placebo, as was performance on several baseline physical function assessments. All participants exhibited low to low–normal total and/or bioavailable testosterone. No notable variance in baseline sex hormone or clinical laboratory values existed between groups (Supplementary Table S1).

**Table 1:** Baseline demographics, spinal cord injury (SCI) characteristics, and functional status.

Variables	All randomized	Vehicle + placebo	TRT + finasteride
	(*N* = 12 M)	(*N* = 5 M)	(*N* = 7 M)
Clinical demographics			
Age, years	60.5 ± 7.8	57.4 ± 9.4	62.7 ± 6.3
Weight, kg	102 ± 23.6	98.1 ± 15.7	104.5 ± 28.9
Height, cm	179 ± 8	174 ± 6	183 ± 7
BMI, kg/m^2^	31.6 ± 6.0	32.6 ± 6.2	31.0 ± 6.2
Ethnicity/race			
Hispanic/Latino, number	2	1	1
Non-Hispanic/Non-Latino, number	10	4	6
Black or African–American, number	3	0	3
White, number	9	5	4
SCI characteristics			
AIS D, *n* (%)	12 (100%)	5 (100%)	7 (100%)
SCI spinal level, range	C2 – L3	C2 – L3	C3 – L2
SCI duration, years	15.5 ± 13.7	21.0 ± 14.7	11.6 ± 10.9
Functional status			
Gait speed, m/s	0.84 ± 0.33	0.76 ± 0.35	0.90 ± 0.33
WISCI II, score	18.9 ± 2.2	19.0 ± 1.7	18.9 ± 2.6
BBS, overall score	45.5 ± 12.3	43.8 ± 17.5	45.8 ± 8.6
BBS score 0–20, *n* (%)	1 (8%)	1 (20%)	0 (0%)
BBS score 21–40, *n* (%)	1 (8%)	0 (0%)	1 (14%)
BBS score 41–56, *n* (%)	10 (83%)	4 (80%)	6 (86%)
SCIM III, score	85.8 ± 14.1	79.2 ± 17.0	90.6 ± 10.4
CHART SF, overall score	464.2 ± 90.8	433.3 ± 112.6	486.2 ± 72.9
Physical independence, score	82.7 ± 33.6	59.2 ± 43.9	99.4 ± 1.51
Cognitive independence, score	80.6 ± 20.9	68.2 ± 24.7	89.4 ± 13.3
Mobility, score	80.9 ± 16.3	78.4 ± 13.9	82.6 ± 18.7
Occupation, score	51.0 ± 40.5	61.9 ± 37.8	43.2 ± 43.3
Social integration, score	78.9 ± 29.0	73.0 ± 36.7	83.1 ± 24.3
Economic self-sufficiency, score	90.1 ± 15.5	92.6 ± 16.5	88.4 ± 15.9
VR-12, physical score	35.5 ± 13.8	28.1 ± 8.7	40.7 ± 14.9
VR-12, mental score	41.3 ± 3.6	43.1 ± 3.3	40.0 ± 3.5
7-Day PAR, estimated kcal/day	3,853 ± 1,136	3,778 ± 1,048	3,906 ± 1,275
SNST, score	7.6 ± 1.1	7.8 ± 1.5	7.4 ± 0.8

Values are mean ± standard deviation (SD), assessed at baseline.

M, males; TRT, testosterone replacement therapy; BMI, body mass index; AIS, ASIA impairment scale; WISCI, Walking Index for Spinal Cord Injury; BBS, Berg Balance Scale; SCIM, Spinal Cord Independence Measure; CHART SF, Craig Handicap Assessment and Reporting Technique Short Form; VR-12, Veteran Rand 12 Item Health Survey; 7-Day PAR, 7-Day Physical Activity Recall; SNST, Spinal Nutrition Screening Tool.

**Table 2:** Baseline body composition, neuromuscular, bone, and prostate values.

Outcome variables	All randomized	Vehicle + placebo	TRT + finasteride
	(*N* = 12 M)	(*N* = 5 M)	(*N* = 7 M)
Body composition outcomes			
Whole-body fat-free mass, kg	65.4 ± 9.9	65.3 ± 6.3	65.4 ± 13
Upper body fat-free mass, kg	44.7 ± 6.2	45.4 ± 5.0	44.2 ± 7.5
Lower body fat-free mass, kg	20.7 ± 4.2	19.9 ± 1.5	21.3 ± 5.7
Whole-body fat mass, kg	30.3 ± 8.6	30.3 ± 9.6	30.4 ± 8.7
Trunk fat mass, kg	16.3 ± 5.4	16.6 ± 6.3	16.0 ± 5.1
Android (visceral) fat mass, kg	3.0 ± 1.3	3.2 ± 1.6	2.9 ± 1.0
Neuromuscular outcomes			
KE whole-muscle CSA, cm^2^	7,054 ± 1783	6,453 ± 1,177	7,483 ± 2096
KE MVIC, Nm	116 ± 35	110 ± 35	121 ± 37
Bone outcomes			
Total hip aBMD, g/cm^2^	0.996 ± 0.171	1.106 ± 0.137	0.979 ± 0.207
Femoral neck aBMD, g/cm^2^	0.852 ± 0.227	0.802 ± 0.152	0.893 ± 0.282
Distal femur aBMD, g/cm^2^	0.802 ± 0.167	0.796 ± 0.131	0.805 ± 0.199
L2-4 aBMD, g/cm^2^	1.190 ± 0.181	1.262 ± 0.153	1.142 ± 0.194
CTX-1, ng/mL	0.38 ± 0.15	0.44 ± 0.20	0.35 ± 0.09
TRAcP 5b, U/L	2.68 ± 0.57	2.58 ± 0.77	2.75 ± 0.44
Osteocalcin, ng/mL	14.4 ± 6.1	14.4 ± 5.0	14.5 ± 7.1
Prostate outcomes			
Prostate volume, mL	22.1 ± 6.4	18.4 ± 5.2	24.8 ± 6.1

Values are mean ± standard deviation (SD), assessed at baseline.

TRT, testosterone replacement therapy; KE, knee extensor; CSA, cross-sectional area; MVIC, maximal voluntary isometric contraction; aBMD, areal bone mineral density; L2-L4, lumbar spine vertebrae 2–4; CTX, type I collagen cross-linked C-telopeptide; TRAcP 5b, tartrate-resistant acid phosphatase 5b (derived from osteoclasts).

### 3.2 Sex-hormone concentrations

**Figure 2:**
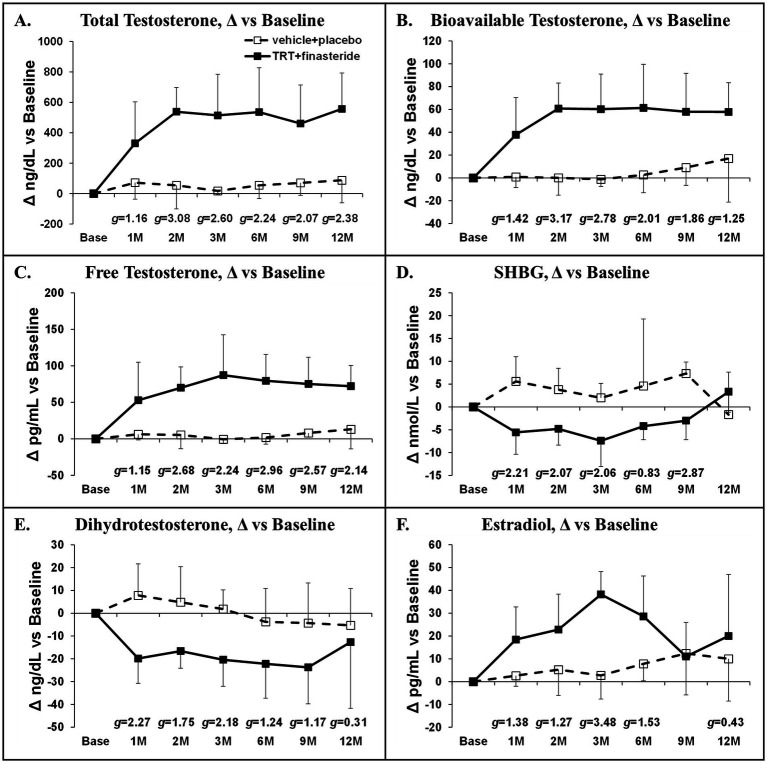
**(A–F)** Absolute sex-steroid hormone changes at 1 month (1 M) through 12 months (12 M) in men who received testosterone replacement therapy plus finasteride (TRT + finasteride, black boxes) or vehicle with placebo (vehicle+placebo, white boxes) after chronic motor-incomplete spinal cord injury (SCI). Values are mean ± standard deviation (SD) of the absolute change vs. baseline, N = 3–7 per group/timepoint. Hedge’s *g* was used to assess effect size [categorized as small (*g* = 0.20), medium (*g* = 0.50), large (*g* = 0.80), or very large (*g* = 1.30)] between groups at each timepoint and reported when values met these thresholds. SHBG, sex-hormone binding globulin.

Baseline total testosterone was 291 ± 135 ng/dL (TRT + finasteride) vs. 280 ± 120 ng/dL (vehicle+placebo). TRT + finasteride produced large to very large effect sizes when compared with vehicle+placebo, suggesting increased total testosterone (+331–557 ng/dL vs. baseline, *g* = 1.16–3.08), increased bioavailable testosterone (+38–61 ng/dL, *g* = 1.25–3.17), and increased free testosterone (+53–87 pg/mL, *g* = 1.15–2.96) at all timepoints (Figures 2A–C). TRT + finasteride also produced small to very large effect sizes indicating reduced SHBG (*g* = 0.83–2.87, Figure 2D) and dihydrotestosterone (*g* = 0.31–2.27, Figure 2E) and increased estradiol (*g* = 0.43–3.48, Figure 2F) at the majority of timepoints vs. vehicle+placebo.

### 3.3 Body composition outcomes

**Figure 3:**
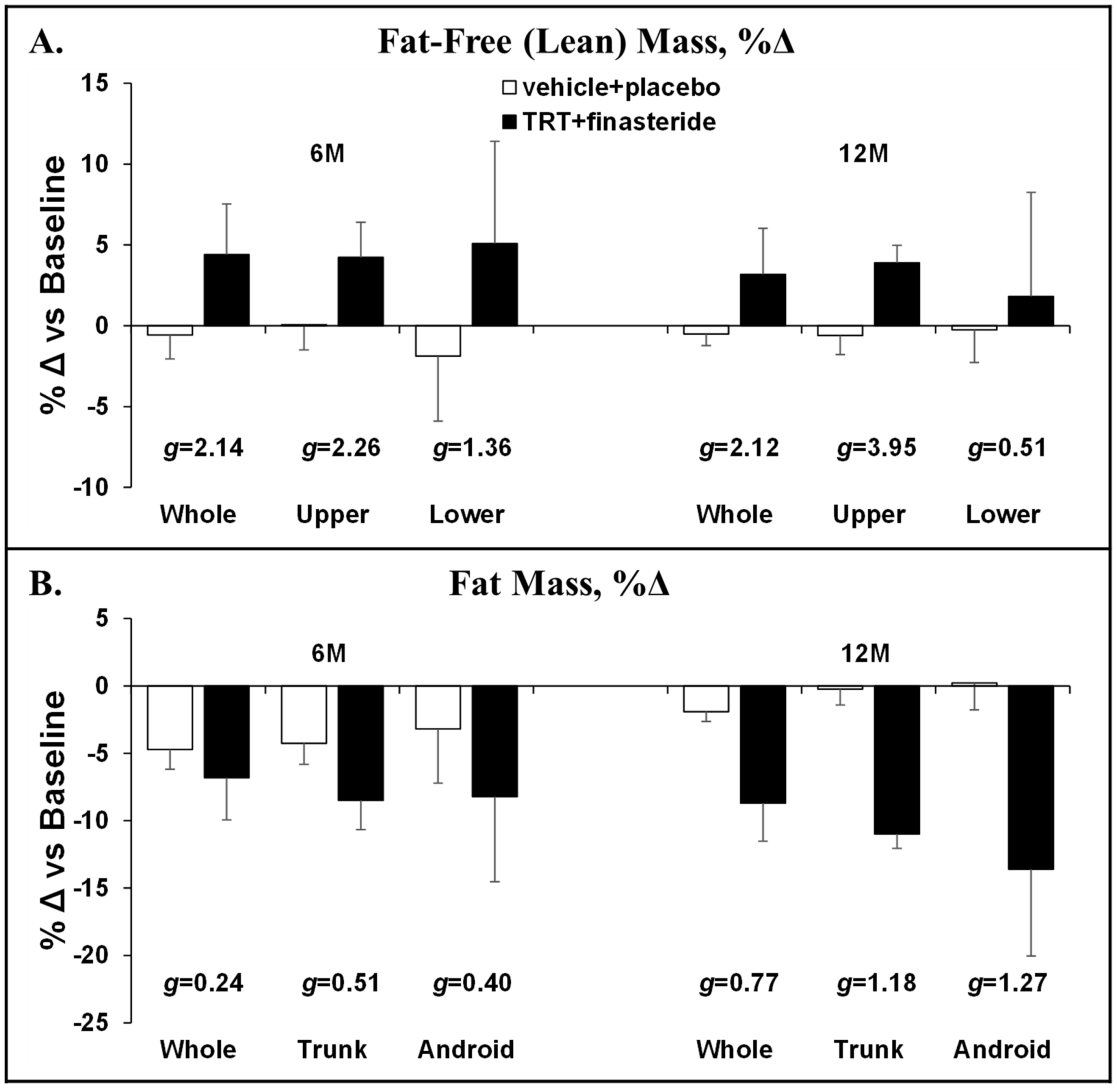
**(A,B)** Percentage change in whole-body and region-specific fat-free mass and fat mass at 6 months (6 M) and 12 months (12 M), derived via dual-energy X-ray absorptiometry (DXA), in men who received testosterone replacement therapy plus finasteride (TRT + finasteride, black bars) or vehicle with placebo (vehicle+placebo, white bars) after chronic motor-incomplete spinal cord injury (SCI). Values are mean ± standard deviation (SD) of the percentage change vs. baseline, *N* = 2–5 per group/timepoint. Hedge’s *g* was used to assess effect size [categorized as small (*g* = 0.20), medium (*g* = 0.50), large (*g* = 0.80), or very large (*g* = 1.30)] between groups at each timepoint and reported when values met these thresholds.

The average change from baseline for whole-body lean mass was +4% and +3% in TRT + finasteride vs. −1% and −1% in vehicle+placebo at 6 and 12 months, respectively (*g* = 2.12–2.14, very large effect sizes; Figure 3A), with the TRT + finasteride group displaying a 4% increase in the upper body lean mass at both the timepoints (*g* = 2.26–3.95, very large effect sizes) and a 2–5% greater increase in lower body lean mass vs. vehicle+placebo (*g* = 0.51–1.36, medium to very large effect sizes). The average change in whole-body fat mass was −7% and −9% in TRT + finasteride vs. −5% and −2% in vehicle+placebo (*g* = 0.24–0.77, small to medium effect sizes; Figure 3B) at 6 and 12 months, respectively, with relatively larger fat loss occurring in the trunk (TRT + finasteride: −8% and −11% vs. vehicle+placebo: −4% and 0%, *g* = 0.51–1.12, medium to large effect sizes) and visceral region (TRT + finasteride: -8% and −14% vs. vehicle+placebo: −3% and 0%, *g* = 0.40–1.27, small to large effect sizes).

### 3.4 Muscular outcomes

**Figure 4:**
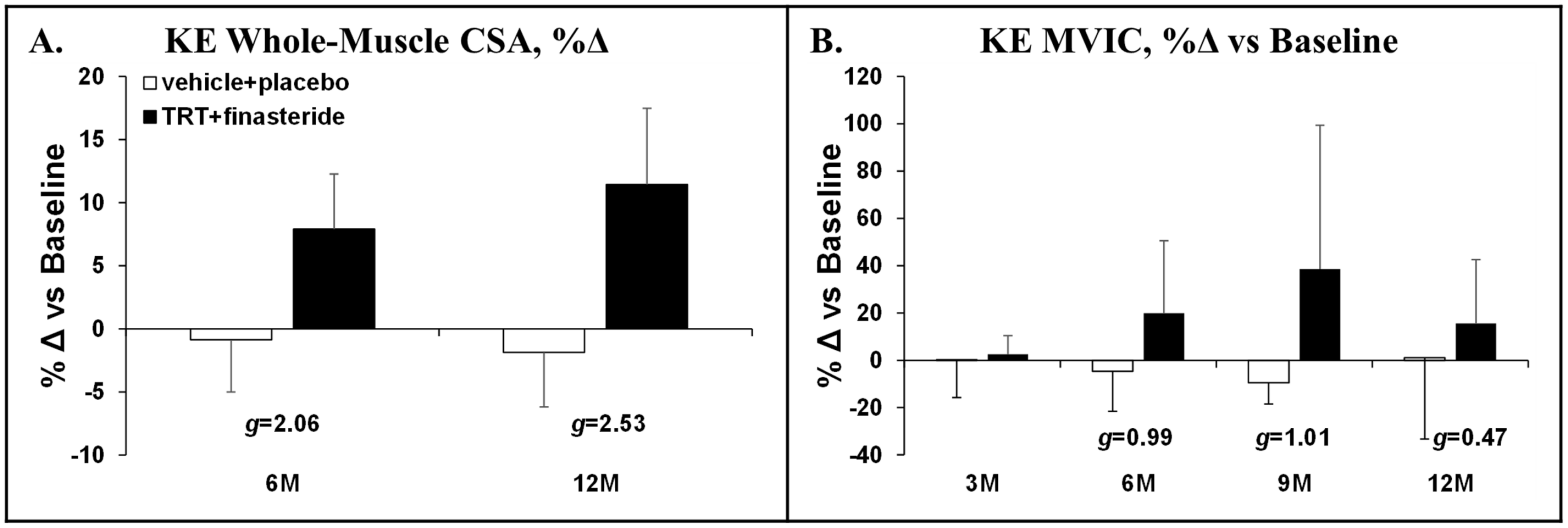
**(A,B)** Percentage change in knee extensors (KE) whole-muscle cross-sectional area (CSA) at 6 months (6M) and 12 months (12M) and KE maximal voluntary isometric contraction (MVIC) at 3 months (3 M) through 12 months (12 M), derived via 3D magnetic resonance imaging (MRI) or dynamometry, respectively, in men who received testosterone replacement therapy plus finasteride (TRT + finasteride, black bars) or vehicle with placebo (vehicle+placebo, white bars) after chronic motor-incomplete spinal cord injury (SCI). Values are mean ± standard deviation (SD) of the percentage change vs. baseline, *N* = 3–5 per group/timepoint. Hedge’s *g* was used to assess effect size [categorized as small (*g* = 0.20), medium (*g* = 0.50), large (*g* = 0.80), or very large (*g* = 1.30)] between groups at each timepoint and reported when values met these thresholds.

The average change for KE whole-muscle CSA was +8% and + 11% in TRT + finasteride vs. −1.0% and − 2.0% in vehicle+placebo at 6 and 12 months, respectively (*g* = 2.06–2.53, very large effect sizes; Figure 4A). This was accompanied by ~15–40% higher KE MVIC in TRT + finasteride from 6 to 12 months vs. −9% to +1% change in vehicle+placebo (*g* = 0.47–1.01, small to large effect sizes; Figure 4B).

### 3.5 Bone outcomes

**Figure 5:**
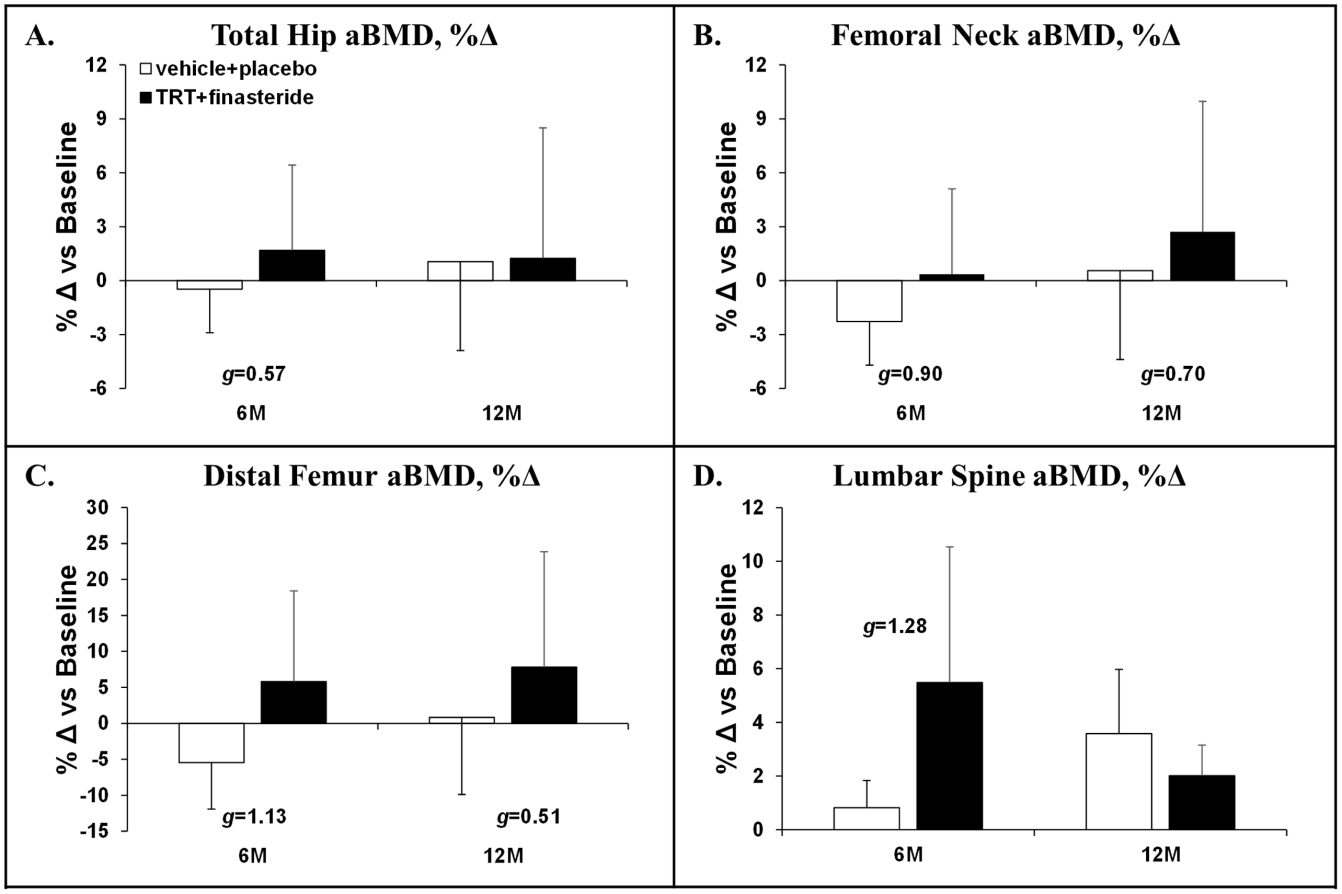
**(A–D)** Percentage change in total hip, femoral neck, distal femur, and lumbar spine (L2–L4) areal bone mineral density (aBMD) at 6 months (6 M) and 12 months (12 M), derived via dual-energy X-ray absorptiometry (DXA), in men who received testosterone replacement therapy plus finasteride (TRT + finasteride, black bars) or vehicle with placebo (vehicle+placebo, white bars) after chronic motor-incomplete spinal cord injury (SCI). Values are mean ± standard deviation (SD) of the percentage change vs. baseline, *N* = 2–5 per group/timepoint. Hedge’s *g* was used to assess effect size [categorized as small (*g* = 0.20), medium (*g* = 0.50), large (*g* = 0.80), or very large (*g* = 1.30)] between groups at each timepoint and reported when values met these thresholds.

**Figure 6:**
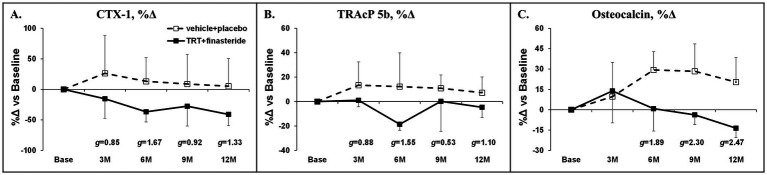
**(A–C)** Percentage change in circulating type I collagen cross-linked C-telopeptide (CTX-1, bone resorption marker), tartrate-resistant acid phosphatase 5b (TRAcP 5b, osteoclast-derived bone resorption marker), and osteocalcin (osteoblast-derived bone formation marker) at 3 months (3 M) through 12 months (12 M) in men who received testosterone replacement therapy plus finasteride (TRT + finasteride, solid lines) or vehicle with placebo (vehicle+placebo, dashed lines) after chronic motor-incomplete spinal cord injury (SCI). Values are mean ± standard deviation (SD) of the percentage change vs. baseline, *N* = 3–5 per group/timepoint. Hedge’s *g* was used to assess effect size [categorized as small (*g* = 0.20), medium (*g* = 0.50), large (*g* = 0.80), or very large (*g* = 1.30)] between groups at each timepoint and reported when values met these thresholds.

The average change for total hip aBMD was +1.7% and + 1.2% in TRT + finasteride vs. −0.5% and + 1.1% in vehicle+placebo at 6 and 12 months, respectively (*g* = 0.57 at 6 months only, medium effect size; Figure 5A), with femoral neck aBMD changing +0.4% and + 2.7% in TRT + finasteride vs. −2.3% and + 0.6% in vehicle+placebo over this period (*g* = 0.70–0.90, medium to large effect sizes; Figure 5B). The distal femur aBMD change from baseline was +6% and + 8% in TRT + finasteride vs. −5% and + 1% in vehicle+placebo at 6 and 12 months (*g* = 0.51–1.13, medium to large effect sizes; Figure 5C), while lumbar spine aBMD increased +5.5% and + 2.0% in TRT + finasteride vs. +0.8% and + 3.6% in vehicle+placebo (*g* = 1.28 at 6 months only, large effect sizes; Figure 5D). The BMD changes were accompanied by reduced CTX-1 and lower TRAcP 5b from 3 to 12 months in TRT + finasteride vs. vehicle+placebo (CTX-1: *g* = 0.85–1.67, large to very large effect sizes; TRAcP 5b: *g* = 0.53–1.55, medium to very large effect sizes; Figures 6A,B), and lower osteocalcin in TRT + finasteride from 6 to 12 months (*g* = 1.89–2.47, very large effect sizes; Figure 6C).

### 3.6 Prostate volume, safety outcomes, and adverse events

The average change from baseline for the prostate volume was 3.2 ± 10.0 mL and 0.4 ± 5.5 mL in TRT + finasteride vs. 5.9 ± 7.1 mL and 3.7 ± 8.5 mL in vehicle+placebo at 6 and 12 months (*g* = 0.31–0.43, small effect sizes), respectively. On average, the values for all *a priori* stopping criteria remained within standard reference ranges in both groups (Supplementary Table S2). One participant in each group was temporarily stopped due to elevated hematocrit and hemoglobin (vehicle+placebo) and elevated liver enzymes (TRT + finasteride), with values resolving at re-testing. Two TRT + finasteride participants were temporarily stopped due to elevated PSA, with values resolving upon re-testing in one and resolving after treatment for lower UTI in the other. The cumulative incidence and severity of all AE were comparable between groups (Supplementary Table S3). However, the incidence of total testosterone, dihydrotestosterone, and estradiol concentrations outside standard reference ranges was more than double in TRT + finasteride vs. vehicle+placebo. Specifically, five of seven TRT + finasteride participants exhibited nadir total testosterone above the reference range upper limit (869 ng/dL), necessitating TRT dose adjustment based on our *a priori* criteria. No mortality events or cardiovascular or cancer-related SAE occurred. One participant in TRT + finasteride was withdrawn for a hospitalization resulting from COVID-19, and one participant in vehicle+placebo was withdrawn after refusing prostate biopsy upon detection of a prostate induration.

### 3.7 Drug compliance and participant retention

Self-reported compliance with TRT or vehicle was 90% vs. 87%, respectively. Finasteride or placebo compliance determined by residual pill counts was 89% vs. 94%, respectively. TRT + finasteride produced anticipated hormonal responses, evidenced by higher total testosterone (+460 ± 115%) and lower dihydrotestosterone (−49 ± 17%) vs. baseline, across timepoints. In comparison, vehicle+placebo produced <25% change for testosterone and increased dihydrotestosterone (+23 ± 21%) vs. baseline. The participant retention was relatively consistent across groups (Supplementary Table S2).

## 4 Discussion

In the 1950s, Cooper et al. published two case series demonstrating that high urinary nitrogen excretion, and a negative nitrogen balance persisted for several months after SCI, and that high-dose intramuscular testosterone (50–100 mg/day) mitigated nitrogen excretion and normalized nitrogen balance in this population (59, 60). As a result, Cooper et al. suggested testosterone treatment may attenuate muscle loss and possibly bone loss after SCI (59, 60). In the 70+ years since these findings, we are aware of only two small prospective clinical trials that assessed musculoskeletal or body composition responses to TRT in men with SCI (34, 35), both being open-label, non-placebo-controlled studies. In the first, Bauman et al. reported that transdermal TRT increased whole-body, trunk, and lower-extremity lean mass, and resting energy expenditure by 7–9% in men (*N* = 11) with chronic motor-complete SCI (34), with improvements persisting 6 months after TRT cessation (61). In contrast, several studies from the second trial indicated that transdermal TRT did not improve body composition or basal metabolic rate (35), nor increase whole-muscle CSA, muscle fiber CSA, electrical-stimulation evoked muscle contractile properties (36–38), or bone microstructural parameters (39) in the paralyzed limbs of men (*N* = 11) with chronic motor-complete SCI. At first, these trials may appear contradictory, although careful examination of each study design provides insight. For example, Bauman et al. (34) administered moderate-dose transdermal TRT (5–10 mg/day; 35–70 mg/week) to men with baseline total testosterone of 250 ± 95 ng/dL, indicating frankly low testosterone. This TRT regimen restored circulating testosterone to the mid-normal physiologic range (432–502 ng/dL) over 6 to 12 months. In comparison, Gorgey et al. (35) provided low-dose transdermal TRT (2–6 mg/day; 14–42 mg/week) for 16 weeks to men whose baseline testosterone was within the eugonadal range on average (431 ± 215 ng/dL) and did not detect any change in circulating testosterone. Collectively, these results demonstrate inconsistency surrounding the impact of TRT on body composition and markers of musculoskeletal health in men with SCI and suggest that higher TRT doses may be required to improve such outcomes after SCI, as has been shown in rodent experimental SCI models (40), and/or that longer TRT durations are needed to observe benefits in persons with SCI.

Herein, we conducted a double-blind, placebo-controlled pilot study that enrolled men with low to low–normal testosterone and ambulatory dysfunction after chronic motor-incomplete SCI and randomized participants to receive TRT + finasteride vs. vehicle+placebo for 12 months. Our focus was to collect preliminary data on efficacy-based body composition, muscle, and bone outcomes that are relevant to the SCI population, while concomitantly assessing prostate size and incidence of AEs that are associated with TRT administration (28). We utilized the broad framework recommended in the Institute of Medicine (IOM) report “Testosterone and Aging: Clinical Research Directions,” (62) which included conducting short-term, double-blind, placebo-controlled RCTs to assess efficacy-based primary outcomes; including participants with testosterone below the physiologic range of young adult men; and ensuring participant safety by including prostate-related exclusion criteria, regular monitoring of PSA, prostate changes, and other AEs. However, our RCT did not specifically enroll older men, as suggested in the IOM report (62), because low testosterone develops at earlier ages in response to SCI (12). However, more than half of our participants were > 60 years of age, and all except one were above the age of 50 years. We evaluated a combinatory regimen that included high-dose intramuscular TRT with finasteride because meta-analyses indicate that intramuscular TRT more distinctly increased lean mass, muscle strength (26), and BMD (27) than transdermal TRT formulations, when compared with their respective placebos, and because several RCTs reported that TRT + finasteride markedly improved body composition, muscle strength, and BMD in older men without neurologic injury (25, 32, 33), while limiting prostate symptoms associated with intramuscular TRT. Herein, we report that TRT + finasteride consistently elevated total testosterone and its bioavailable and free testosterone subfractions (large to very large effect sizes throughout), while concomitantly reducing dihydrotestosterone (large to very large effect sizes) at the majority of the timepoints. From an efficacy standpoint, TRT + finasteride produced moderate to very large ES at both 6 and 12 months for several efficacy outcomes, including (1) whole-body, upper body, and lower body lean mass, (2) trunk fat mass, (3) KE whole-muscle CSA, and (4) femoral neck and distal femur aBMD. In addition, moderate to very large effect sizes were detected at several distinct timepoints from 6 months onward for whole-body and visceral fat mass, KE MVIC, and all other efficacy-based outcomes in response to TRT + finasteride. From a safety standpoint, small effect sizes favoring smaller prostate increases in TRT + finasteride vs. vehicle+placebo were present at 6 and 12 months. Moreover, no differences in the incidence of AEs commonly associated with TRT existed between groups (28).

Intramuscular TRT dose-dependently increases circulating testosterone and its bioactive metabolites—dihydrotestosterone and estradiol (63)—via localized tissue-specific actions of the 5α-reductase and aromatase enzymes (64), respectively. In our study, intramuscular TRT increased circulating testosterone into the mid-to-high physiologic range and also reduced SHBG, similar to a previous RCT that utilized an identical TRT dose (65), which produced higher bioavailable (non-SHBG-bound) and free (unbound) testosterone. TRT + finasteride also increased estradiol and reduced dihydrotestosterone by 50–60%, like previous RCTs that utilized this combinatory drug regimen (25, 32, 33). Our study design cannot distinguish whether the SHBG and estradiol changes were solely due to high-dose TRT or whether these changes were influenced by finasteride co-administration. However, Amory et al. reported no changes in SHBG and estradiol in men receiving finasteride or dutasteride, a dual-type I/II 5α-reductase inhibitor, over 12 months (66) and our laboratory previously reported that older men receiving TRT alone exhibited increased estradiol and that finasteride co-administration did not alter this response (25), suggesting these changes likely resulted primarily from TRT with little influence from finasteride. Interestingly, previous RCTs have shown that higher TRT doses produced greater muscular benefit in older men without neurologic injury and that neither 5α-reductase (25, 32, 67) nor aromatase (68) activity is required for muscle gain. Furthermore, the conversion of testosterone to dihydrotestosterone via the type II 5α-reductase enzyme is not required for the lipolytic or BMD benefits of TRT in older men, but this conversion exacerbates prostate growth associated with TRT (25, 33). In comparison, the aromatization of testosterone partially mediates the TRT-induced regulation of bone metabolism (69, 70), BMD (71), and lipolysis in men (68). As such, the increases in testosterone and estradiol and the simultaneous reduction in dihydrotestosterone resulting from TRT + finasteride likely underlie our results.

We observed a 4–5% greater increase in whole-body lean mass in TRT + finasteride vs. vehicle+placebo at both 6 and 12 months (very large effect sizes at both), which is comparable to that occurring in hypogonadal older men without neurologic injury who received TRT + finasteride (25). This lean mass gain was relatively consistent in the upper body and impaired lower extremities, similar to the findings of Bauman et al. (34), and was accompanied by 8–11% higher KE whole-muscle CSA (very large effect sizes throughout) and a > 15% increase in KE MVIC at 6 months onward (moderate to large effect sizes), similar to that observed in other RCTs that administered intramuscular TRT to older men without neurologic injury (23–25). Notably, prior research indicates that the preservation and/or restoration of KE strength is a key predictor of walking function in persons with motor-incomplete SCI (72), providing a rationale to assess gait parameters in future TRT studies conducted in the incomplete SCI population. In comparison, Gorgey et al. reported that low-dose transdermal TRT did not increase circulating testosterone (35) nor increase lean mass, muscle CSA, or electrical stimulation-evoked muscle force production in the paralyzed limbs of men with chronic complete SCI over 4 months (36–38). Given this, our data appear to be the first direct evidence to suggest that TRT produces muscular benefit in men with chronic SCI, when given in a sufficient dose and duration.

TRT is known to reduce visceral fat mass in men with central adiposity (73). We observed a greater reduction in adiposity in TRT + finasteride vs. vehicle+placebo, which was most evident in the trunk and visceral depot that displayed ~11–14% greater fat mass reduction at 12 months (large effect sizes for both). These reductions in whole-body and trunk fat were similar in magnitude to previous RCTs administering TRT + finasteride in older men without neurologic injury (25, 32). While we did not investigate the mechanisms underlying the lipolytic effects of TRT, this likely relied on the aromatization of testosterone (68). For example, Holland et al. reported that high-dose testosterone-enanthate reversed visceral fat accumulation in orchiectomized rats through an estrogen-dependent mechanism that suppressed several genes that influence fat accumulation, including lipoprotein lipase and fatty acid synthase (74). Alternatively, it is possible that fat loss resulting from TRT may be influenced by increased resting and/or activity-dependent energy expenditure. In this regard, Bauman et al. reported that TRT increased resting energy expenditure by ~8% or ~ 115 kcal/day in men with motor-complete SCI (34) and preclinical studies demonstrate that testosterone treatment restored voluntary physical activity in orchiectomized rodents in a manner that relied on aromatase (75, 76). Moreover, estradiol is known to promote the recovery of respiratory function and improve respiratory neuroplasticity in the rodent C2-hemisection model, which has the potential to indirectly benefit physical function (77). As such, our results provide a rationale to discern the mechanism(s) through which TRT + finasteride stimulates fat loss after SCI and to determine whether a visceral fat reduction of the magnitude observed herein is sufficient to improve cardiometabolic health in persons with chronic SCI, especially given that high visceral fat mass predicts increased cardiometabolic risk in persons with chronic SCI (7, 78) and that low testosterone is associated with a harmful cardiometabolic profile in this population (4).

Increased bone resorption and/or reduced bone formation after SCI leads to imbalanced bone turnover and progressive cancellous and cortical bone loss (2), contributing to the 100-fold higher lower-extremity fracture risk in men with SCI at age 50, compared to the general population (79). In older men without neurologic injury, TRT increases hip and lumbar spine BMD (80), when provided in a sufficient dose and duration. However, only one small exploratory study has assessed the skeletal responses to TRT in persons with SCI, reporting that 16 weeks of low-dose transdermal TRT did not improve MRI-derived bone microstructural parameters at the proximal, mid-diaphyseal, or distal femur (39). In comparison, we observed a 6–8% increase in distal femur aBMD at 6 and 12 months (medium to large effect sizes at both) in response to TRT + finasteride, along with hip and spine aBMD changes that were comparable to previous RCTs administering TRT + finasteride to older men (25, 33). The differences in skeletal responses between the Holman et al. exploratory study (39) and our findings are likely due to differences in study duration and design (16-weeks non-placebo controlled RCT vs. 12 months double-blind, placebo-controlled RCT) or to differences in TRT modality and dose (transdermal TRT, 2–6 mg/day or 14–42 mg/week vs. intramuscular TRT, 125 mg/week) that influence the circulating testosterone and/or estradiol responses. Indeed, in rodent SCI models, the bone responses to TRT are dose-dependent, with higher doses producing more profound skeletal benefit (40), and may be influenced by the aromatization to estradiol, as has been shown in adult men (64). The aBMD improvements we observed were accompanied by lower circulating CTX-1, TRAcP 5b (bone resorption markers), and osteocalcin (bone formation marker) in TRT + finasteride (moderate to very large effect sizes), consistent with reduced bone turnover due to the known antiresorptive actions of testosterone (70). Given the high incidence of distal femur fractures following SCI (2), we are encouraged by the distal femur aBMD increase, which was larger in magnitude and more consistent than BMD changes at the hip, possibly due to the high concentration of trabeculae at the distal femur. In this regard, Snyder et al. reported that TRT increased volumetric BMD more so in trabecular bone compartments vs. cortical-rich peripheral bone, across several skeletal sites, in older hypogonadal men (80). Similarly, in a rodent SCI model, we have shown that high-dose intramuscular testosterone-enanthate prevented trabecular bone loss at several key sublesional sites, including the femoral neck, distal femur, and proximal tibia, but did not impact cortical bone at these sites (40–42). In line with this speculation, over the initial 6 months of the study, TRT + finasteride increased aBMD at the lumbar spine, which is an area rich in trabecular bone, although these apparent changes reverted by 12 months, likely due to participant withdrawals in both groups over the final 6 months. As such, future studies assessing TRT-induced skeletal changes should incorporate imaging modalities that can distinguish trabecular and cortical bone compartments with those that can predict bone mechanical characteristics in response to torsional and compressive loading, such as computerized tomography with finite element analysis, along with more standard DXA measurements (81), to ensure a more complete understanding of the impact of TRT on bone structure and strength.

TRT is associated with increased risk for several side effects, including polycythemia, a small HDL cholesterol reduction, and combined prostate/lower urinary tract-related events, including increased PSA, prostate enlargement, and prostate nodules necessitating biopsy (28). However, a complex relationship exists between these side effects and the change in the sex-hormone milieu after TRT, with estradiol serving as a better predictor of the hemoglobin and HDL changes than testosterone or dihydrotestosterone (82). Given this, it is essential to mention that our TRT + finasteride regimen occasionally increased testosterone above the physiologic range and produced highly elevated estradiol, necessitating downward TRT dose adjustments in some participants. Regardless, TRT + finasteride produced no incidence of polycythemia, defined in our study as hematocrit >52% or hemoglobin >17.5 g/dL. The increased polycythemia risk in men receiving TRT has raised concern regarding potential cardiovascular risks, particularly in older men with mobility limitations (83). For example, the Testosterone in Older Men with Sarcopenia (TOM) Trial, which enrolled older men with low testosterone and mobility limitations not resulting from neuromuscular disease, was discontinued based on recommendations of the Data Safety Monitoring Board due to a higher incidence of cardiovascular-related AEs in the testosterone vs. placebo group (83). Although a recent multisite RCT that evaluated 5,000+ hypogonadal men with pre-existing cardiovascular risk factors reported that TRT did not increase major adverse cardiovascular events over a 4-years follow-up, when compared with placebo (84). Additionally, TRT + finasteride produced negligible PSA change and a lesser prostate size increase than vehicle+placebo (small effect sizes), along with no prostate nodules or indurations, confirming previous studies that report TRT + finasteride may produce lower prostate risk than TRT-alone (25, 32, 33). This remains important in the SCI population because testosterone is independently associated with prostate size in men with SCI (85) and because benign prostate hyperplasia may produce complications when assessing neurogenic bladder issues common in men with SCI (86) and may limit ease of intermittent catheterization (87).

As with all studies, several limitations warrant discussion. First, we enrolled men with low to low–normal testosterone and ambulatory dysfunction after chronic motor-incomplete SCI resulting from trauma, vascular, or orthopedic pathology. As such, our results should not be generalized to other populations, such as those with SCI due to differing pathology, those with acute or subacute SCI or complete (AIS A/B) SCI, or men with normal testosterone after SCI. Second, while we comprehensively assessed sex-steroid hormones, including total testosterone and its subfractions, estradiol, dihydrotestosterone, and SHBG, we did not assess gonadotropins, so we cannot discern whether low testosterone was due to primary or secondary causes. Third, this study was terminated prior to achieving our *a priori* enrollment target, in part, due to the emergence of the SARS-CoV-2 (COVID-19) pandemic and the resulting nationwide VA-mandated halt on in-person research visits. Given this, randomization resulted in somewhat differing baseline KE whole-muscle CSA, KE MVIC, and prostate volume values between groups. As such, our results are considered preliminary and require confirmation. However, this pilot study fulfilled its primary purpose, which was to elicit preliminary data on TRT + finasteride efficacy in the chronic incomplete SCI population that can be targeted for confirmation in larger, more comprehensive clinical trials.

Based on our findings, we suggest several future directions to advance TRT-related research within the SCI population. First, large, well-controlled RCTs are needed to validate the observations reported herein. When designing such studies, we suggest (1) enrolling men with chronic incomplete SCI who exhibit unequivocally low testosterone and signs and/or symptoms of hypogonadism and that meet other Endocrine Society (88) and Society for Endocrinology (89) recommendations to ensure appropriateness of testosterone therapy, (2) employing a multisite approach to increase likelihood of meeting recruitment goals, (3) utilizing a study duration of 12 months or longer to ensure sufficient time exists to detect musculoskeletal and body composition changes, (4) incorporating an intramuscular TRT titration protocol to ensure circulating testosterone does not exceed the physiologic range for adult men, and (5) following framework in the IOM report on “Testosterone and Aging: Clinical Research Directions” concerning the assessment of efficacy and safety outcomes (62). Second, it remains essential to determine the potential clinical relevance of TRT-induced body composition and musculoskeletal changes. Several vital questions that warrant investigation, include: Are body composition changes of the magnitude reported herein sufficient to impact cardiometabolic health? Do TRT-induced muscular adaptations improve physical function and/or quality of life or reduce frailty or disability status in those with motor-incomplete SCI? Are BMD changes that result from TRT sufficient to increase bone strength or reduce fracture risk after SCI, particularly in the highly fracture-prone regions that surround the knee? Third, studies are needed to assess whether intramuscular TRT lessens muscle and bone loss throughout the acute to subacute post-SCI recovery period when the majority of the men exhibit low testosterone (8, 9), especially considering that high-dose intramuscular testosterone treatment has been shown to mitigate nitrogen excretion and normalize nitrogen balance in the weeks to months after SCI (59, 60). Fourth, it is essential to discern whether intramuscular TRT improves muscle and bone integrity and body composition in persons with complete SCI, given that these individuals typically display more severe muscle and bone loss and higher fracture risk than those with incomplete SCI and that no pharmacologic has shown consistent success in improving musculoskeletal integrity after complete SCI (1, 2). In this regard, assessing the impact of intramuscular TRT with and without activity-based physical rehabilitation or other therapeutic modalities that impart mechanical strains in the impaired limbs would assist in understanding the unique interplay between the nervous, endocrine, and musculoskeletal systems during paralysis (90). Finally, from a mechanistic perspective, assessing the individual and combined effects of intramuscular TRT and finasteride is necessary to impart an understanding of how each drug impacts safety and efficacy in this population and to better discern the influence(s) of testosterone and dihydrotestosterone on tissues of interest.

In summary, we report findings from the first double-blind, placebo-controlled RCT assessing TRT efficacy in persons with SCI. The effect sizes detected herein suggest TRT + finasteride increased circulating testosterone and estradiol into the high physiologic range, while concomitantly reducing dihydrotestosterone, which was the underlying premise of this study. From an efficacy standpoint, TRT + finasteride produced large to very large effect sizes for key body composition, muscle, and bone outcomes at both 6 and 12 months, in a magnitude similar to that occurring in older hypogonadal men without neurologic injury in response to intramuscular TRT (25, 32), and for all other efficacy outcomes at select timepoints after 6 months. These data suggest that a minimum 6-months regimen of high-dose intramuscular TRT may be required to improve body composition and musculoskeletal integrity in the SCI population. From a safety standpoint, small effect sizes favored lesser prostate growth in TRT + finasteride, with similar AE rates detected in both groups, suggesting that known prostate-related side effects of high-dose TRT may be minimized by finasteride co-administration, as has been observed in other populations (25, 32, 33). Collectively, these findings provide proof-of-concept and rationale for future RCTs focused on determining the ability of intramuscular TRT with finasteride to safely improve body composition, musculoskeletal health, and physical function in men with low testosterone and impaired gait after SCI, along with effect sizes and variability data to assist in planning such trials.

## Data Availability

The datasets presented in this article are not readily available because the datasets generated in this study are property of the US Government. Datasets will be made available in a de-identified, anonymized manner upon written request per US Department of Veterans Affairs policy and in accordance with the Freedom of Information Act. Requests to access the datasets should be directed to Joshua.Yarrow@va.gov.
