# Supplementary material for: Musculoskeletal and body composition response to high-dose testosterone with finasteride after chronic incomplete spinal cord injury—a randomized, double-blind, and placebo-controlled pilot study
Source: Front Neurol. 2024 Dec 11;15:1479264. doi: 10.3389/fneur.2024.1479264 (PMC11668665; doi:10.3389/fneur.2024.1479264)
Supplement: Supplementary file 1 [file Table_1.docx]

Supplementary Material

| **Supplementary Table 1.** Baseline sex-steroid hormone concentrations, complete blood count, comprehensive metabolic and lipid panels, and other clinical laboratory values. | | | | |
| --- | --- | --- | --- | --- |
| **Baseline Clinical Lab Values** | **Lab Reference Range** | **All Randomized**  **(N=12 M)** | **Vehicle + Placebo**  **(N=5 M)** | **TRT + Finasteride**  **(N=7 M)** |
| **Sex-Steroid Hormones** | | | | |
| **Total testosterone, ng/dL** | 193 – 869 | 287 ± 124 | 280 ± 120 | 291 ± 135 |
| **Bioavailable testosterone, ng/dL** | N/A | 52 ± 16 | 55 ± 11 | 50 ± 19 |
| **Free testosterone, pg/mL** | 46 – 224 ^a^ | 38 ± 17 | 36 ± 15 | 39 ± 19 |
| **Dihydrotestosterone, ng/dL** | 16 – 79 | 29 ± 16 | 25 ± 18 | 32 ± 15 |
| **Estradiol, pg/mL** | ≤ 29 | 23 ± 13 | 19 ± 9 | 26 ± 15 |
| **SHBG, nmol/L** | 10 – 50 | 37 ± 13 | 33 ± 14 | 40 ± 12 |
| **Complete Blood Count (CBC)** | | | | |
| **WBC, k/cmm** | 4.2 – 10.3 | 7.04 ± 1.50 | 7.47 ± 1.41 | 6.72 ± 1.59 |
| **RBC, M/cmm** | 4.44 – 6.1 | 4.70 ± 0.53 | 4.34 ± 0.54 | 4.95 ± 0.38 |
| **Hemoglobin, g/dL** | 13.9 – 17.0 | 14.1 ± 1.4 | 13.5 ± 1.6 | 14.5 ± 1.2 |
| **Hematocrit, %** | 41 – 52 | 41.7 ± 4.2 | 39.5 ± 5.0 | 43.3 ± 3.0 |
| **MCV, um^3^** | 80 – 98 | 89.0 ± 4.5 | 91.1 ± 5.3 | 87.6 ± 3.6 |
| **MCH, pg** | 27.0 – 33.3 | 30.1 ± 1.6 | 31.2 ± 1.5 | 29.3 ± 1.1 |
| **MCHC, g/dL** | 31.8 – 37.1 | 33.9 ± 1.2 | 34.4 ± 1.5 | 33.5 ± 0.9 |
| **Platelets, k/cmm** | 130 – 440 | 227 ± 40 | 219 ± 32 | 233 ± 47 |
| **RDW-SD, fL** | 39.0 – 52.2 | 42.5 ± 3.6 | 43.2 ± 3.7 | 42.0 ± 3.8 |
| **RDW, %** | 11.3 – 16.5 | 13.1 ± 0.9 | 12.9 ± 0.5 | 13.2 ± 1.2 |
| **MPV, um^3^** | 7.4 – 10.5 | 10.2 ± 0.5 | 10.0 ± 0.6 | 10.4 ± 0.4 |
| **Granulocytes #, k/cmm** | 1.8 – 7.8 | 4.12 ± 1.14 | 4.47 ± 1.13 | 3.87 ± 1.16 |
| **Lymphocytes #, k/cmm** | 1.1 – 3.4 | 2.03 ± 0.56 | 2.11 ± 0.68 | 1.99 ± 0.51 |
| **Monocytes #, k/cmm** | 0.14 – 0.76 | 0.62 ± 0.17 | 0.64 ± 0.12 | 0.60 ± 0.21 |
| **Eosinophils #, k/cmm** | 0.1 – 0.5 | 0.20 ± 0.11 | 0.18 ± 0.06 | 0.22 ± 0.13 |
| **Basophils #, k/cmm** | 0.0 – 0.1 | 0.04 ± 0.02 | 0.05 ± 0.02 | 0.03 ± 0.02 |
| **Immature granulocytes #, k/cmm** | 0.0 – 0.2 | 0.02 ± 0.01 | 0.03 ± 0.02 | 0.02 ± 0.01 |
| **Granulocytes, %** | 54 – 65 | 58.0 ± 7.4 | 59.5 ± 8.4 | 56.9 ± 7.0 |
| **Lymphocytes, %** | 12 – 45 | 29.3 ± 6.7 | 28.3 ± 8.1 | 29.9 ± 6.1 |
| **Monocytes, %** | 3 – 7 | 8.8 ± 1.8 | 8.8 ± 2.2 | 8.8 ± 1.7 |
| **Eosinophils, %** | 0.0 – 6.0 | 3.0 ± 1.8 | 2.4 ± 0.6 | 3.5 ± 2.3 |
| **Basophils, %** | 0.0 – 2.0 | 0.6 ± 0.3 | 0.7 ± 0.3 | 0.5 ± 0.3 |
| **Immature granulocytes, %** | 0.0 – 0.6 | 0.4 ± 0.2 | 0.4 ± 0.2 | 0.3 ± 0.1 |
| **NRBC, #** | 0 – 0.2 | 0.00 ± 0.00 | 0.00 ± 0.00 | 0.00 ± 0.00 |
| **NRBC, per 100 WBC** | 0 – 6 | 0 ± 0 | 0 ± 0 | 0 ± 0 |
| **Comprehensive Metabolic Panel** | | | | |
| **Glucose, mg/dL** | 65 – 99 | 104 ± 28 | 89 ± 23 | 116 ± 28 |
| **Urea nitrogen, mg/dL** | 9 – 20 | 17 ± 8 | 21 ± 11 | 14 ± 4 |
| **Creatinine, mg/dL** | 0.5 – 1.2 | 1.1 ± 0.3 | 1.2 ± 0.4 | 1.0 ± 0.2 |
| **eGFR-IDMS, mL/min** | > 60 | 58 ± 5 ^b^ | 56 ± 7 ^b^ | > 60 ± 0.0 |
| **Sodium, mmol/L** | 135 – 145 | 140 ± 1.4 | 139 ± 1.5 | 141 ± 1.3 |
| **Potassium, mmol/L** | 3.5 – 5.0 | 4.3 ± 0.4 | 4.5 ± 0.5 | 4.2 ± 0.3 |
| **Chloride, mmol/L** | 98 – 108 | 101 ± 1.7 | 101 ± 1.3 | 102 ± 2.0 |
| **CO_2_, mmol/L** | 23 – 32 | 26 ± 1.4 | 26 ± 1.6 | 26 ± 1.3 |
| **Anion gap, mmol/L** | 5 – 15 | 13 ± 1.6 | 12 ± 0.7 | 13 ± 1.9 |
| **Calcium, mg/dL** | 8.4 – 10.5 | 9.7 ± 0.3 | 9.7 ± 0.3 | 9.7 ± 0.2 |
| **Protein, total, g/dL** | 6.0 – 8.2 | 7.5 ± 0.5 | 7.2 ± 0.4 | 7.8 ± 0.3 |
| **Albumin, g/dL** | 3.5 – 5.0 | 4.5 ± 0.1 | 4.5 ± 0.1 | 4.5 ± 0.2 |
| **Bilirubin, total, mg/dL** | 0.0 – 1.3 | 0.4 ± 0.2 | 0.4 ± 0.1 | 0.4 ± 0.2 |
| **Alkaline phosphatase, U/L** | 0 – 125 | 85 ± 23 | 78 ± 22 | 90 ± 23 |
| **ALT, U/L** | 0 – 40 | 25 ± 6 | 25 ± 3 | 25 ± 7 |
| **AST, U/L** | 0 – 45 | 24 ± 7 | 27 ± 9 | 21 ± 6 |
| **Lipid Panel** | | | | |
| **Cholesterol, total, mg/dL** | 0 – 199 | 184 ± 45 | 168 ± 24 | 196 ± 54 |
| **Cholesterol, HDL, mg/dL** | ≥ 40 | 49 ± 18 | 52 ± 24 | 47 ± 13 |
| **Cholesterol, LDL, mg/dL** | ≤ 129 | 100 ± 39 | 80 ± 18 | 113 ± 45 |
| **Triglycerides, mg/dL** | 0 – 149 | 168 ± 121 | 179 ± 127 | 161 ± 127 |
| **Other Lab Tests** | | | | |
| **CRP, mg/dL** | < 0.1 – 0.4 | 0.4 ± 0.3 | 0.3 ± 0.2 | 0.5 ± 0.3 |
| **Erythropoietin, mIU/mL** | 2.6 – 18.5 | 11.5 ± 4.2 | 13.7 ± 3.3 | 9.9 ± 4.2 |
| **Hemoglobin (Hb)A1c, %** | < 5.7% | 5.8 ± 0.9 | 5.5 ± 0.7 | 5.9 ± 0.9 |
| **Insulin, uIU/mL** | ≤ 19.6 | 16.1 ± 20.4 ^c^ | 25.8 ± 30.3 ^c^ | 9.1 ± 3.4 |
| **PSA, ng/mL** | 0 – 4.0 | 0.99 ± 0.53 | 0.85 ± 0.61 | 1.09 ± 0.50 |
| Values are Mean ± SD, assessed at baseline. TRT = testosterone replacement therapy, N/A = not available, SHBG = sex-hormone binding globulin, WBC = white blood count, RBC = red blood count, MCV = mean corpuscular volume, MCH = mean corpuscular hemoglobin, MCHC = mean corpuscular hemoglobin concentration, RDW-SD = red cell distribution width-standard deviation, RDW = red cell distribution width, MPV = mean platelet volume, NRBC = nucleated red blood cell, eGFR-IDMS = estimated glomerular filtration rate-isotope dilution mass spectroscopy, CO_2_ = carbon dioxide, ALT = alanine transaminase, AST = aspartate aminotransferase, HDL = high-density lipoprotein, LDL = low-density lipoprotein, CRP = c-reactive protein, PSA = prostate-specific antigen.  ^a^ Free testosterone reference range for males 18 – 69 years of age, which encompassed N=11 of 12 participants. Reference range is 6 – 73 pg/mL for males 70 – 89 years of age, which encompassed N = 1 of 12 participants.  ^b^ N=2 individuals had eGFR-IDMS <60 mL/min, all others were >60 mL/min and were assigned a value = 60 to calculate values.  ^c^ N=1 individual was insulin dependent diabetic. | | | | |
