# Supplementary material for: Musculoskeletal and body composition response to high-dose testosterone with finasteride after chronic incomplete spinal cord injury—a randomized, double-blind, and placebo-controlled pilot study
Source: Front Neurol. 2024 Dec 11;15:1479264. doi: 10.3389/fneur.2024.1479264 (PMC11668665; doi:10.3389/fneur.2024.1479264)
Supplement: Supplementary file 2 [file Table_2.docx]

Supplementary Material

| **Supplementary Table 2.** Participant retention and laboratory values for all *a priori* stopping criteria in men who received testosterone replacement therapy (TRT) plus finasteride or vehicle with placebo after chronic motor-incomplete spinal cord injury (SCI). | | | | | | | |
| --- | --- | --- | --- | --- | --- | --- | --- |
|  | **Baseline** | **1M** | **2M** | **3M** | **6M** | **9M** | **12M** |
| **Participant Retention, N remaining/enrolled (%)** | | | | | | | |
| **Vehicle + Placebo** | 5/5 (100%) | 5/5 (100%) | 5/5 (100%) | 5/5 (100%) | 5/5 (100%) | 3/5 (60%) | 3/5 (60%) |
| **TRT + Finasteride** | 7/7 (100%) | 7/7 (100%) | 6/7 (86%) | 5/7 (71%) | 5/7 (71%) | 4/7 (57%) | 3/7 (43%) |
| **ALT, U/L** | | | | | | | |
| **Vehicle + Placebo** | 25 ± 3 | 29 ± 11 | 28 ± 7 | 24 ± 5 | 25 ± 3 | 27 ± 4 | 28 ± 7 |
| **TRT + Finasteride** | 25 ± 7 | 23 ± 9 | 32 ± 21 | 21 ± 8 | 20 ± 6 | 20 ± 4 | 22 ± 2 |
| **AST, U/L** | | | | | | | |
| **Vehicle + Placebo** | 27 ± 9 | 24 ± 4 | 26 ± 4 | 23 ± 4 | 27 ± 6 | 21 ± 2 | 21 ± 4 |
| **TRT + Finasteride** | 21 ± 6 | 22 ± 4 | 36 ± 30 | 21 ± 6 | 23 ± 8 | 22 ± 1 | 28 ± 13 |
| **Calcium, mg/dL** | | | | | | | |
| **Vehicle + Placebo** | 9.7 ± 0.3 | 9.5 ± 0.2 | 9.7 ± 0.4 | 9.6 ± 0.3 | 9.5 ± 0.5 | 10.1 ± 0.7 | 9.6 ± 0.5 |
| **TRT + Finasteride** | 9.7 ± 0.2 | 9.5 ± 0.4 | 9.3 ± 0.3 | 9.6 ± 0.6 | 9.6 ± 0.7 | 9.9 ± 0.4 | 9.5 ± 0.3 |
| **Hematocrit, %** | | | | | | | |
| **Vehicle + Placebo** | 39.5 ± 5.0 | 40.6 ± 4.7 | 41.4 ± 4.4 | 42.1 ± 6.9 | 40.9 ± 6.0 | 40.9 ± 5.9 | 40.5 ± 4.4 |
| **TRT + Finasteride** | 43.3 ± 3.0 | 44.1 ± 3.7 | 45.3 ± 4.4 | 45.3 ± 4.0 | 46.7 ± 3.3 | 46.2 ± 2.8 | 45.1 ± 3.0 |
| **Hemoglobin, g/dL** | | | | | | | |
| **Vehicle + Placebo** | 13.5 ± 1.6 | 14.0 ± 1.6 | 14.2 ± 1.4 | 14.5 ± 2.3 | 13.9 ± 2.1 | 13.6 ± 1.7 | 13.4 ± 1.2 |
| **TRT + Finasteride** | 14.5 ± 1.2 | 14.4 ± 1.4 | 14.7 ± 1.6 | 14.7 ± 1.6 | 15.4 ± 1.5 | 15.2 ± 1.3 | 14.7 ± 1.5 |
| **PSA, ng/mL** | | | | | | | |
| **Vehicle + Placebo** | 0.85 ± 0.61 | 0.77 ± 0.37 | 0.84 ± 0.43 | 0.79 ± 0.47 | 0.74 ± 0.49 | 0.79 ± 0.70 | 0.69 ± 0.61 |
| **TRT + Finasteride** | 1.09 ± 0.50 | 0.98 ± 0.48 | 1.55 ± 1.33 | 1.34 ± 1.05 | 1.22 ± 1.02 | 0.89 ± 0.52 | 1.04 ± 0.62 |
| **Prostate Volume, mL** | | | | | | | |
| **Vehicle + Placebo** | 18.4 ± 5.2 | N/A | N/A | N/A | 24.3 ± 11.6 | N/A | 25.0 ± 12.3 |
| **TRT + Finasteride** | 24.8 ± 6.1 | N/A | N/A | N/A | 26.1 ± 12.2 | N/A | 25.9 ± 6.9 |
| Values are Mean ± SD, N=3-7 per group/timepoint. ALT = alanine transaminase, AST = aspartate aminotransferase, PSA = prostate-specific antigen, N/A = not assessed. | | | | | | | |
