# Supplementary material for: Musculoskeletal and body composition response to high-dose testosterone with finasteride after chronic incomplete spinal cord injury—a randomized, double-blind, and placebo-controlled pilot study
Source: Front Neurol. 2024 Dec 11;15:1479264. doi: 10.3389/fneur.2024.1479264 (PMC11668665; doi:10.3389/fneur.2024.1479264)
Supplement: Supplementary file 3 [file Table_3.docx]

Supplementary Material

| **Supplementary Table 3.** Incidence of serious adverse events (SAE), non-serious adverse events (AE) that were *a priori* stopping criteria, and other non-serious AE categorized by system in men who received testosterone replacement therapy plus finasteride (TRT+FIN) or matching vehicle with placebo after chronic motor-incomplete spinal cord injury (SCI). | | | |
| --- | --- | --- | --- |
| **Adverse Event Category** | **All Randomized**  **(N=12 M)** | **Vehicle + Placebo**  **(N=5 M)** | **TRT + Finasteride**  **(N=7 M)** |
| **Serious Adverse Events (SAE)** | | | |
| **All-cause mortality, # (events)** | 0/12 (0) | 0/5 (0) | 0/7 (0) |
| **Myocardial ischemia, # (events)** | 0/12 (0) | 0/5 (0) | 0/7 (0) |
| **Acute ischemic event, # (events)** | 0/12 (0) | 0/5 (0) | 0/7 (0) |
| **Acute coronary syndrome, # (events)** | 0/12 (0) | 0/5 (0) | 0/7 (0) |
| **Congestive heart failure, # (events)** | 0/12 (0) | 0/5 (0) | 0/7 (0) |
| **Flow limiting CAD, # (events)** | 0/12 (0) | 0/5 (0) | 0/7 (0) |
| **Cancer (all-cause), # (events)** | 0/12 (0) | 0/5 (0) | 0/7 (0) |
| **Hospitalization (cardiac), # (events)** | 0/12 (0) | 0/5 (0) | 0/7 (0) |
| **Hospitalization (all-cause), # (events)** | 1/12 (1) | 0/5 (0) | 1/7 (1) **^a^** |
| **Other SAE, # (events)** | 0/12 (0) | 0/5 (0) | 0/7 (0) |
| **Non-Serious Adverse Events – Stopping Criteria** | | | |
| **ALT or AST >1.5X upper limit, # (events)** | 1/12 (2) | 0/5 (0) | 1/7 (2) ^b^ |
| **Calcium >11.2 mg/dL or 10.5 – 11.2 mg/dL**  **with hypercalcemia symptoms, # (events)** | 0/12 (0) | 0/5 (0) | 0/7 (0) |
| **Hematocrit >52%, # (events)** | 1/12 (1) | 1/5 (1) ^c^ | 0/7 (0) |
| **Hemoglobin >17.5 g/dL, # (events)** | 1/12 (1) | 1/5 (1) ^c^ | 0/7 (0) |
| **Gynecomastia, # (events)** | 0/12 (0) | 0/5 (0) | 0/7 (0) |
| **Peripheral edema ≥ 2+, # (events)** | 0/12 (0) | 0/5 (0) | 0/7 (0) |
| **Prostate nodule/induration, # (events)** | 1/12 (1) | 1/5 (1) ^d^ | 0/7 (0) |
| **PSA >4.0 ng/mL or increase >1.4 ng/mL,**  **# (events)** | 2/12 (3) | 0/5 (0) | 2/7 (3) ^e^ |
| **Other Non-Serious Adverse Events (AE)** | | | |
| **Ear disorders** | | | |
| **otitis media, # (events)** | 1/12 (1) | 0/5 (0) | 1/7 (1) |
| **Eye disorders** | | | |
| **eye infection, # (events)** | 1/12 (1) | 1/5 (1) | 0/7 (0) |
| **General disorders** | | | |
| **aldolase (H), # (events)** | 1/12 (1) | 0/5 (0) | 1/7 (1) |
| **ALT (H), # (events)** | 2/12 (2) | 1/5 (1) ^f^ | 1/7 (1) ^f^ |
| **anion gap (H), # (events)** | 3/12 (4) | 2/5 (3) | 1/7 (1) |
| **albumin (H), # (events)** | 1/12 (1) | 1/5 (1) | 0/7 (0) |
| **alkaline phosphatase, # (events)** | 1/12 (4) | 1/5 (4) | 0/7 (0) |
| **calcium (H), # (events)** | 2/12 (3) | 1/5 (1) ^f^ | 1/7 (2) ^f^ |
| **chloride (L), # (events)** | 1/12 (1) | 1/5 (1) | 0/7 (0) |
| **cholesterol HDL (L), # (events)** | 7/12 (27) | 2/5 (13) | 5/7 (14) ^g^ |
| **cholesterol LDL (H), # (events)** | 3/12 (12) | 0/5 (0) | 3/7 (12) ^h^ |
| **cholesterol total (H), # (events)** | 6/12 (19) | 2/5 (5) | 4/7 (14) ^i^ |
| **CO_2_ (L), # (events)** | 3/12 (8) | 1/5 (1) | 2/7 (7) |
| **CPK (H), # (events)** | 1/12 (3) | 0/5 (1) | 1/7 (2) |
| **creatinine (H), # (events)** | 4/12 (5) | 2/5 (2) | 2/7 (3) |
| **CRP (H), # (events)** | 9/12 (27) | 3/5 (9) ^j^ | 6/7 (18) ^j^ |
| **dihydrotestosterone (H), # (events)** | 1/12 (2) | 1/5 (2) | 0/7 (0) |
| **dihydrotestosterone (L), # (events)** | 7/12 (21) | 2/5 (8) | 5/7 (13) |
| **edema (<2+), # (events)** | 1/12 (2) | 0/5 (0) | 1/7 (2) |
| **eosinophils (H), # (events)** | 10/12 (36) | 5/5 (12) | 5/7 (22) |
| **erythropoietin (H), # (events)** | 4/12 (14) | 2/5 (5) | 2/7 (9) |
| **estradiol (H), # (events)** | 11/12 (35) | 4/5 (7) | 7/7 (28) |
| **glucose (H), # (events)** | 12/12 (52) | 5/5 (23) ^k^ | 7/7 (29) ^k^ |
| **granulocytes (H), # (events)** | 5/12 (16) | 4/5 (12) | 1/7 (4) |
| **granulocytes (L), # (events)** | 7/12 (19) | 3/5 (8) | 4/7 (11) |
| **hematocrit (L), # (events)** | 4/12 (21) | 3/5 (17) | 1/7 (4) |
| **hemoglobin (L), # (events)** | 5/12 (24) | 3/5 (15) | 2/7 (9) |
| **insulin (H), # (events)** | 6/12 (22) | 4/5 (20) | 2/7 (2) |
| **potassium (H), # (events)** | 1/12 (1) | 0/5 (0) | 1/7 (1) |
| **potassium (L), # (events)** | 2/12 (2) | 2/5 (2) | 0/7 (0) |
| **lymphocytes (H), # (events)** | 5/12 (13) | 2/5 (6) | 3/7 (7) |
| **lymphocytes (L), # (events)** | 7/12 (28) | 4/5 (17) | 3/7 (11) |
| **MCH (L), # (events)** | 2/12 (7) | 0/5 (0) | 2/7 (7) |
| **MCHC (L), # (events)** | 4/12 (9) | 1/5 (1) | 3/7 (8) |
| **MCV (H), # (events)** | 1/12 (1) | 1/5 (1) | 0/7 (0) |
| **MCV (L), # (events)** | 1/12 (5) | 0/5 (0) | 1/7 (5) |
| **monocytes (H), # (events)** | 12/12 (57) | 5/5 (23) ^l^ | 7/7 (34) ^l^ |
| **MPV (H), # (events)** | 7/12 (10) | 3/5 (5) | 4/7 (5) |
| **myelocytes (H), # (events)** | 1/12 (1) | 0/5 (0) | 1/7 (1) |
| **total protein (H), # (events)** | 2/12 (2) | 1/5 (1) | 1/7 (1) |
| **RBC (L), # (events)** | 5/12 (15) | 3/5 (12) | 2/7 (3) |
| **RDW (H), # (events)** | 3/12 (9) | 0/5 (0) | 3/7 (9) |
| **RDW (L), # (events)** | 2/12 (6) | 2/5 (6) | 0/7 (0) |
| **segmented neutrophils (L), # (events)** | 1/12 (1) | 0/5 (0) | 1/7 (1) |
| **SHBG (H), # (events)** | 1/12 (3) | 1/5 (3) | 0/7 (0) |
| **SHBG (L), # (events)** | 1/12 (5) | 1/5 (5) | 0/7 (0) |
| **sodium (H), # (events)** | 1/12 (1) | 1/5 (1) | 0/7 (0) |
| **total testosterone (H), # (events)** | 5/12 (12) | 0/5 (0) | 5/7 (12) ^m^ |
| **total testosterone (L), # (events)** | 3/12 (9) | 1/5 (7) | 2/7 (2) ^n^ |
| **triglycerides (H), # (events)** | 7/12 (28) | 3/5 (17) ^o^ | 4/7 (11) ^o^ |
| **urea nitrogen (H), # (events)** | 2/12 (2) | 2/5 (2) | 0/7 (0) |
| **urea nitrogen (L), # (events)** | 2/12 (4) | 0/5 (0) | 2/7 (4) |
| **urobilinogen (H), # (events)** | 1/12 (1) | 0/5 (0) | 1/7 (1) |
| **WBC (H), # (events)** | 2/12 (2) | 1/5 (1) | 1/7 (1) |
| **WBC (L), # (events)** | 1/12 (1) | 0/5 (0) | 1/7 (1) |
| **Musculoskeletal and connective tissue disorders** | | | |
| **falls, # (events)** | 3/12 (5) | 3/5 (5) ^p^ | 0/7 (0) |
| **musculoskeletal pain, # (events)** | 6/12 (9) | 3/5 (5) ^p^ | 3/7 (4) ^p^ |
| **Nervous system disorders** | | | |
| **dysarthria, # (events)** | 1/12 (1) | 1/5 (1) | 0/7 (0) |
| **paresthesia, # (events)** | 1/12 (1) | 1/5 (1) | 0/7 (0) |
| **Psychiatric disorders** | | | |
| **depression, # (events)** | 1/12 (1) | 0/5 (0) | 1/7 (1) ^p^ |
| **Renal and urinary disorders** | | | |
| **lower urinary tract infection, # (events)** | 1/12 (1) | 0/5 (0) | 1/7 (2) |
| **Reproductive system disorders** | | | |
| **impaired ejaculation, # (events)** | 1/12 (2) | 1/5 (2) ^p^ | 0/7 (0) |
| **penile shrinkage, # (events)** | 1/12 (1) | 0/5 (0) | 1/7 (1) ^p^ |
| **testicular atrophy, # (events)** | 1/12 (1) | 0/5 (0) | 1/7 (1) ^p^ |
| Values = participants affected/enrolled and (cumulative events). Data on SAE and AE were collected via systemic assessment during regular study visits at baseline and at 1-, 2-, 3-, 6-, 9-, and 12-months of participation and via self-report during weekly telephone assessments for actively enrolled participants. AE were considered any lab derived values outside the standard reference range, using a 5% frequency threshold for reporting non-serious AE within each group. CAD = coronary artery disease, PSA = prostate-specific antigen, H = high (above standard reference range), L = low (below standard reference range), AST = aspartate aminotransferase, ALT = alanine transaminase, HDL = high-density lipoprotein, LDL = low-density lipoprotein, CO_2_ = carbon dioxide, CPK = creatine phosphokinase, CRP = C-reactive protein, MCH = mean corpuscular hemoglobin, MCHC = mean corpuscular hemoglobin concentration, MCV = mean corpuscular volume, MPV = mean platelet volume, RBC = red blood cell count, RDW = red blood cell distribution width, SHBG = sex hormone binding globulin, WBC = white blood cell count.  ^a^ Hospitalization due to severe acute respiratory syndrome (SARS)-CoV-2 (COVID-19) infection, participant was withdrawn due to SAE.  ^b^ Values resolved upon re-testing, participant was not withdrawn.  ^c^ Value resolved upon re-testing, participant was not withdrawn.  ^d^ Prostate induration was noted upon digital rectal exam, participant refused prostate biopsy and was withdrawn due to *a priori* stopping criteria.  ^e^ Lower urinary tract infection detected twice in 1 participant and treatment was provided, PSA renormalized with each treatment, participant was not withdrawn. PSA renormalized in 1 participant within 1-month, participant was not withdrawn.  ^f^ Values were above standard reference range but did not exceed *a priori* stopping criteria.  ^g^ 3 of 5 TRT + finasteride participants with low HDL cholesterol exhibited values below standard reference range at baseline (before treatment initiation).  ^h^ 3 of 3 TRT + finasteride participants with high LDL cholesterol exhibited values above standard reference range at baseline (before treatment initiation).  ^i^ 4 of 4 TRT + finasteride participants with high total cholesterol exhibited values above standard reference range at baseline (before treatment initiation).  ^j^ 3 of 6 TRT + finasteride participants and 1 of 3 vehicle + placebo participants with high CRP exhibited values above standard reference range at baseline (before treatment initiation).  ^k^ 7 of 7 TRT + finasteride participants and 3 of 5 vehicle + placebo participants with high glucose exhibited values above standard reference range at baseline (before treatment initiation).  ^l^ 6 of 7 TRT + finasteride participants and 3 of 5 vehicle + placebo participants with high monocytes exhibited values above standard reference range at baseline (before treatment initiation).  ^m^ >869 ng/dL, values were retested and TRT dose adjusted per protocol.  ^n^ <193 ng/dL, values were retested and TRT dose adjusted per protocol.  ^o^ 3 of 4 TRT + finasteride participants and 3 of 3 vehicle + placebo participants with high triglycerides exhibited values above standard reference range at baseline (before treatment initiation).  ^p^ Self-reported AE, not adjudicated. | | | |
